# The *osa‐miR164* target *OsCUC1* functions redundantly with *OsCUC3* in controlling rice meristem/organ boundary specification

**DOI:** 10.1111/nph.16939

**Published:** 2020-10-25

**Authors:** Jun Wang, Jinlin Bao, Beibei Zhou, Min Li, Xizhi Li, Jian Jin

**Affiliations:** ^1^ State Key Laboratory for Conservation and Utilization of Subtropical Agro‐bioresources College of Life Science and Technology Guangxi University Nanning 530005 China

**Keywords:** boundary specification, CLD1, degradation, *miR164*, partially redundant, *CUP‐SHAPED COTYLEDON* genes, twisted‐rolling leaves

## Abstract

- The specification of the meristem/organ boundary is critical for plant development. Here, we investigate two previously uncharacterized NAC transcription factors: the first, OsCUC1, which is negatively regulated by *osa‐miR164c*, dimerizes with the second, OsCUC3, and functions partially redundantly in meristem/organ boundary specification in rice (*Oryza sativa*).
- We produced knockout lines for rice *OsCUC1* (the homolog of Arabidopsis *CUC1* and *CUC2*) and *OsCUC3* (the homolog of Arabidopsis *CUC3*), as well as an overexpression line for *osa‐miR164c,* to study the molecular mechanism of boundary specification in rice.
- A single mutation in either *OsCUC1* or *OsCUC3* leads to defects in the establishment of the meristem/organ boundary, resulting in reduced stamen numbers and the fusion of leaves and filaments, and the defects are greatly enhanced in the double mutant. Transgenic plants overexpressing *osa‐miR164c* showed a phenotype similar to that of the *OsCUC1* knockout line. In addition, knockout of *OsCUC1* leads to multiple defects, including dwarf plant architecture, male sterility and twisted‐rolling leaves. Further study indicated that OsCUC1 physically interacts with leaf‐rolling related protein CURLED LEAF AND DWARF 1 (CLD1) and stabilizes it in the nucleus to control leaf morphology.
- This work demonstrated that the interplay of *osa‐miR164c*, *OsCUC1* and *OsCUC3* controls boundary specification in rice.

## Introduction

Most of the aerial parts of higher plants are derived from the shoot apical meristem (SAM). In the SAM, the creation of a boundary that separates a meristem/organ primordium from its surroundings is critical for organ formation. In Arabidopsis, the *CUP‐SHAPED COTYLEDON* (*CUC*) genes *CUC1* and *CUC2*, which encode a paralogous pair of NAC transcription factors and are negatively regulated by the microRNA *miR164*, have been shown to be involved in embryonic SAM formation and boundary specification (Aida & Tasaka, 2006). Since *CUC1* and *CUC2* are functionally redundant, neither the *cuc1* nor *cuc2* single mutant displays a severe seedling phenotype, while the *cuc1 cuc2* double mutant shows severe cotyledon fusion and the absence of a SAM (Aida *et al*., 1997, 1999; Takada *et al*., 2001). Another NAC gene family member, *CUC3*, which is a homolog of *CUC1* and *CUC2*, also participates in the establishment of the cotyledon boundary and the shoot meristem redundantly with *CUC1* and *CUC2* (Vroemen *et al*., 2003; Hibara *et al*., 2006).

Systematic sequence analysis revealed 151 putative NAC or NAC‐like genes in rice (*Oryza sativa* L.) (Nuruzzaman *et al*., 2010). Some of these genes have been demonstrated to be involved in different rice developmental processes. For example, *NAC29/30* regulates cellulose synthesis (Huang *et al*., 2015); *O. sativa NAC‐like activated by apetala3/pistillata* (*OsNAP*) positively regulates leaf senescence and serves as a link between abscisic acid (ABA) and leaf senescence (Chen *et al*., 2014; Liang *et al*., 2014); *OsNAC2* not only affects plant height, shoot branching, and thus, yield (Mao *et al*., 2007; Chen *et al*., 2015; Jiang *et al*., 2018), but also promotes leaf senescence via ABA biosynthesis (Mao *et al*., 2017); ONAC020, ONAC023 and ONAC026 heteromerize and play important roles in seed development (Mathew *et al*., 2016). In addition, many rice NAC genes have been shown to be involved in responses to various abiotic and biotic stresses, including *ONAC048* (*OsNAC6*), *ONAC017* (*OsNAC111*), *ONAC122*, *ONAC131*, *ONAC054* (*RICE DWARF VIRUS MULTIPLICATION 1*) and *ONAC068* (*OsNAC4*) in defense against pathogen infection (Nakashima *et al*., 2007; Kaneda *et al*., 2009; Yoshii *et al*., 2009; Sun *et al*., 2012; Yokotani *et al*., 2014), and *ONAC022*, *ONAC002* (*STRESS‐RESPONSIVE NAC 1*/*OsNAC9*), *ONAC048* (*SNAC2*/*OsNAC6*), *ONAC009* (*OsNAC5*), *ONAC122* (*OsNAC10*), *ONAC045*, *ONAC058* (*OsNAP*), *ONAC004*, *ONAC060*, *ONAC011* and *ONAC104* in abiotic stress tolerance (Hu *et al*., 2006, 2008; Nakashima *et al*., 2007; Zheng *et al*., 2009; Jeong *et al*., 2010, 2013; Song *et al*., 2011; Redillas *et al*., 2012; Chen *et al*., 2014; Fang *et al*., 2014; Liang *et al*., 2014; Hong *et al*., 2016).

Although many NAC genes have been characterized in rice, whether they are involved in meristem or organ boundary specification remains unknown. To clarify this question, we knocked out the homologues of *CUC* genes in rice using CRISPR/Cas9. Our results indicated that *OsCUC1*, which is negatively regulated by *osa‐miR164c*, together with *OsCUC3*, is involved in rice meristem/organ boundary specification in a partially redundant manner. In addition, OsCUC1 physically interacts with a previously known leaf rolling related protein CURLED LEAF AND DWARF 1 (CLD1) and stabilizes it in the nucleus to control leaf morphology.

## Materials and Methods

### Plant materials and growth conditions

Rice (*Oryza sativa* L.) plants were grown in a glasshouse at 30°C (for daytime)/25°C (for nighttime) under a 13.5 h : 8.5 h, light : dark photoperiod (>3000 lux) with 60% humidity. The genetic background of all transgenic plants used was Nipponbare. *omtn4* ^ZH11^ and *omtn6* ^ZH11^ were obtained from Biogle GeneTech (Hangzhou Biogle Co. Ltd, Hangzhou, Zhejiang, China)

### Phylogenetic tree and sequence alignment

Rice OsCUC1, OsCUC3 and 70 homologues from 13 species were chosen for phylogenetic analysis. Protein sequences were obtained from NCBI (https://www.ncbi.nlm.nih.gov/) according to their accession number. The sequence alignment was performed with clustalx and the phylogenetic tree was constructed using mega7 with the neighbor‐joining method and bootstrap analysis (1000 replicates). The accession numbers are listed in Supporting Information Table S1.

### Vector construction

To knock out *OsCUC1* and *OsCUC3*, target sites were designed online (http://cbi.hzau.edu.cn/crispr/), and the optimal target sites with low off‐target scores and high sgRNA scores were selected. We prepared the CRISPR/Cas9 binary constructs as described previously (Ma *et al*., 2015). To overexpress *osa‐miR164c*, a polymerase chain reaction (PCR) fragment amplified from Nipponbare genomic DNA using primers OE‐miR164cF and OE‐miR164cR was cloned into the PstI/SpeI sites of binary vector *pOX* between the maize *Ubi1* promoter and Nos terminators.

Generation of *pOsCUC1::GUS* was achieved by amplifying a 2 kb *OsCUC1* promoter from Nipponbare genomic DNA using primers OsCUC1pgus‐F and OsCUC1pgus‐R, which was then inserted into *pCAMBIA1300‐GN* at the HindIII/XbaI site. To generate *pOsCUC1::OsCUC1‐GUS*, a PCR fragment harboring the 2 kb upstream promoter and gDNA (without a stop codon) of *OsCUC1* was amplified from Nipponbare genomic DNA using primers pro‐cds‐gus‐F and pro‐cds‐gus‐R, and was then inserted into *pCAMBIA1300‐GN* at the SalI/XbaI site. To generate *pOsCUC1::mOsCUC1‐GUS*, two overlapped fragments harboring the 2 kb upstream promoter and gDNA (without a stop codon) of *OsCUC1* were amplified using two primer sets: pro‐cds‐gus‐F/mOsCUC1‐R (carrying the 7 bp mutations) and mOsCUC1‐F (carrying the 7 bp mutations)/pro‐cds‐gus‐R, respectively. After purification, the two fragments were mixed in equal molar ratios and used as the PCR templates to amplify a fragment containing the 2 kb upstream promoter and gDNA (without a stop codon) of *OsCUC1*, into which the *osa‐miR164c*‐resistant mutation was introduced, using pro‐cds‐gus‐F and pro‐cds‐gus‐R. After sequencing, the fragment was inserted into *pCAMBIA1300‐GN* at the SalI/XbaI site.

Primer sequences for the constructions are listed in Table S2.

### Pollen viability test

Mature pollen grains from the unopened flowers were collected and immediately put on a microscope slide. A drop of IKI (iodine potassium iodide) solution was deposited onto the pollen, and the slide was covered with a coverslip. Pollen viability counts were made 5 min after the IKI solution was added to the pollen. Pollen grains exhibiting dark staining (dark red or black color) were counted as viable.

### Histochemical analysis and β‐glucuronidase (GUS) assay

Samples of the transgenic plants were incubated with GUS staining solution (50 mM sodium phosphate at pH 7.2, 10 mM ethylenediaminetetraacetic acid (EDTA), 0.1% Triton X‐100, 2 mM of X‐Gluc, 2 mM potassium ferricyanide, 2 mM potassium ferrocyanide) overnight at 37°C. After staining, the tissues were rinsed several times with ethanol, then mounted on slides and photographed.

For histochemical analysis, the samples were fixed with formaldehyde alcohol acetic acid (FAA) fixation solution at 4°C overnight, followed by dehydration and embedding in paraffin (Paraplast Plus, Sigma). They were then cut into 7 μm sections with a microtome, and stained with safranine and fast green FCF. Sections were observed under bright field with a microscope (CX31; Olympus, Tokyo, Japan).

### Yeast‐two‐hybrid assay

The Matchmaker yeast‐two‐hybrid system (Clontech, Kusatsu, Japan) was used to study the interaction of OsCUC1, OsCUC3, OMTN4 and OMTN6 with CLD1. The deduced amino acid sequences of OsCUC1, OsCUC3, OMTN4 and OMTN6 were separately cloned into *pGADT7* (*AD*) vectors, while CLD1 was inserted into *pGBKT7* (*BD*). Yeast strain Y2HGold was transformed with bait plasmid and strain Y187 was transformed with prey plasmid. Co‐transformants were plated on synthetic defined (SD)/–Leu/–Trp/–His/–Ade medium plates and SD/–Leu/–Trp/–His/–Ade/X‐α‐Gal medium plates for examination of growth. Primer sequences for the constructions are listed in Table S2.

### Transactivation assay

The deduced amino acid sequences of OsCUC1 and OsCUC3 were separately cloned into *pGBKT7* (*BD*), resulting in fusions with the GAL4 binding domain. The fusion plasmids *pBD‐OsCUC1* and *pBD‐OsCUC3* were transformed into yeast strain AH109 and plated on SD/–Trp/–His/–Ade and SD/–Trp/–His/–Ade/X‐α‐Gal medium plates for examination of growth. Primer sequences for the constructions are listed in Table S2.

### Scanning electron microscopy (SEM)

The samples were fixed in FAA (50% ethanol/acetic acid/formaldehyde, 9 : 0.5 : 0.5), and then dehydrated in an ethanol series and dried by supercritical fluid drying with CO_2_. The dried samples were mounted on copper supports and sputter‐coated with gold, and then observed under SEM (S‐3400N; Hitachi, Tokyo, Japan).

### Bi‐molecular fluorescence complementation (BiFC) assay

To study the interactions of OsCUC1, OsCUC3, OMTN4, OMTN6, CLD1, CUC1 CUC2, CUC3, mCUC1 and mCUC2, the coding sequences of these genes were correspondingly cloned into *p35S‐Vn* and *p35S‐Vc*. The plasmids were co‐expressed in rice/Arabidopsis protoplasts. The protein–protein interaction was evaluated using a confocal laser scanning microscope (TCS‐SP8MP; Leica, Wetzlar, Germany). Primer sequences for the constructions are listed in Table S2.

### Subcellular localization assay

The coding sequences of *OsCUC1*, *OsCUC3* and *CLD1* (without stop codons) from Nipponbare were fused with green fluorescent protein (GFP) to generate the fusion protein. The fusion protein driven by the cauliflower mosaic virus (CaMV) 35S promoter was transcribed in rice protoplasts. The subcellular localization was evaluated using a confocal laser‐scanning microscope (TCS‐SP8MP; Leica). OsRac3‐mCherry and Ghd7‐mCherry were used as the membrane localization marker (Chen *et al*., 2010) and nuclear localization marker (Xue *et al*., 2008), respectively. Primer sequences for the constructions are listed in Table S2.

### RNA extraction and quantitative real time (RT) polymerase chain reaction assay

To evaluate gene expression, total RNA was extracted using the RNeasy Plant Mini Kit (cat. no. 74904; Qiagen, Germany) following the manufacturer's instructions. First‐strand cDNA was synthesized from 1 μg of total RNA using the PrimeScript RT Reagent Kit (cat. no. RR047A; Takara, Kusatsu, Japan) according to the manufacturer’s instructions. ChamQ SYBR qPCR Master Mix (cat. no. Q311‐01; Vazyme, Nanjing, Jiangsu, China) and a LightCycler 480II (Roche) were used for qRT‐PCR, according to the manufacturers’ instructions. Rice *Ubiquitin* (*Os03g0234200*) was used as the internal reference, and the level of gene expression was normalized to the *Ubiquitin* level. To evaluate the expression level of *osa‐miR164*, RNA extraction and stem‐loop qRT‐PCR were performed as described previously (Wu *et al*., 2007). Rice miRNA *U6* was used as the internal reference and the *osa‐miR164* level was normalized to *U6*. Primer sequences for the qRT‐PCR are listed in Table S2.

### *In situ* hybridization

To prepare the probe, a 500‐bp fragment of *OsCUC1*‐specific cDNA and a 500‐bp fragment of *OsCUC3*‐specific cDNA were amplified. The probes were labeled using a DIG RNA Labeling Kit (Roche). The *in situ* hybridization experiments were carried out as described previously (Brewer *et al*., 2006). Primer sequences for the *in situ* hybridization are listed in Table S2.

### Co‐immunoprecipitation (Co‐IP) assay

To confirm the protein–protein interactions of OsCUC1, OsCUC3 and CLD1, *OsCUC1‐GFP*, *OsCUC1‐MYC*, *OsCUC3‐GFP* and *CLD1‐Flag* were artificially synthesized (GenScript Biotech Corp., Nanjing, Jiangsu, China) and transcribed under the control of the cauliflower mosaic virus (CaMV) 35S promoter. Twelve hours after co‐transformation of the plasmids, the rice protoplasts were harvested by centrifugation at 400 ***g*** for 5 min. The collected protoplasts were homogenized in CelLytic immunoprecipitation (IP) buffer (B7345; Sigma‐Aldrich), and incubated for 30 min on ice and then centrifuged at 15 000 ***g*** for 10 min at 4°C to remove aggregates. Next, 40 μl of Protein A/G Agarose Beads (nProtein A Sepharose 4 Fast Flow; GE Healthcare, Uppsala, Sweden) were added into the protein extract, which was diluted by IP buffer. The mixture was incubated for 3 h at 4°C with gentle shaking (40–50 rpm). After centrifugation at 14 000 ***g*** for 5 min at 4°C, the supernatant was separated from the beads, and the antibodies were added to the supernatant. After overnight incubation at 4°C, 40 μl of protein A‐Sepharose was added and incubated for a further 2–3 h at 4°C. The beads were collected by centrifugation at 100 ***g*** for 3 min at 4 °C, and then washed five times with ice‐cold washing buffer (25 mM Tris‐HCl, pH 7.5, 150 mM NaCl, 0.5% Triton X‐100, 1 mM EDTA, and 1% protease inhibitor). The proteins were eluted from the beads by boiling in sodium dodecyl sulfate–polyacrylamide gel electrophoresis (SDS‐PAGE) sample buffer for 5 min and analysed by Western blotting. The original blot images are shown in Fig. S1. Primer sequences for the constructions are listed in Table S2.

### *In vitro* pull‐down assay

To confirm the protein–protein interaction of OsCUC1 and OsCUC3, the coding sequences of *OsCUC1* and *OsCUC3* were amplified and inserted into *pGEX‐6P‐1* (in frame fusion with a glutathione‐*S*‐transferase (GST) tag) and *pRSFDuet‐MBP* (in frame fusion with a maltose binding protein (MBP) tag), respectively, and then *GST‐OsCUC1* and *MBP‐OsCUC3* were amplified and cloned into *pT_N_T* (Promega). The recombinant GST‐OsCUC1 and MBP‐OsCUC3 were synthesized using the TNT SP6 High‐Yield Wheat Germ Protein Expression System (Promega) following the manufacturer’s instructions. The recombinant GST‐OsCUC1 fusion protein was immobilized on GST‐Binding Resin (MagneGST Glutathione Particles, Promega) and incubated with MBP‐OsCUC3 for 4 h at 4°C. After incubation, the beads were washed five times in washing buffer (4.2 mM Na_2_HPO_4_, 2 mM KH_2_PO_4_, 140 mM NaCl and 10 mM KCl), and subsequently eluted using elution buffer (50 mM Tris‐HCl, 50 mM glutathione). The supernatant was subjected to immunoblotting analysis with anti‐MBP (TransGen, Beijing, China) and anti‐GST (TransGen) antibodies.

To confirm the protein–protein interaction of OsCUC1 and CLD1, the coding sequence of *CLD1* was amplified and inserted into *pRSFDuet‐His* (in frame fusion with a His tag), and then *His‐CLD1* was amplified and cloned into *pT_N_T* (Promega). The recombinant His‐CLD1 was synthesized using the TNT SP6 High‐Yield Wheat Germ Protein Expression System (Promega) following the manufacturer’s instructions. The recombinant GST‐OsCUC1 fusion protein was immobilized on GST‐Binding Resin (MagneGST™ Glutathione Particles; Promega) and incubated with His‐CLD1 for 4 h at 4°C. After incubation, the beads were washed five times in washing buffer (4.2 mM Na_2_HPO_4_, 2 mM KH_2_PO_4_, 140 mM NaCl and 10 mM KCl), and subsequently eluted using elution buffer (50 mM Tris‐HCl, 50 mM glutathione). The supernatant was subjected to immunoblotting analysis with anti‐His (TransGen) and anti‐GST (TransGen) antibodies.

The original blot images are shown in Fig. S1. Primer sequences for the constructions are listed in Table S2.

### Protein extraction

For an *in planta* CLD1 accumulation assay, total proteins were extracted from 0.2 g of 12‐d‐old fresh leaves of non‐KO1*^OsCUC1^* and *oscuc1*‐KO1. The leaves were collected and ground in liquid nitrogen, then homogenized in protein extraction buffer (50 mM Tris‐HCl, pH7.5, 150 mM NaCl, 4 M urea and 1 mM phenylmethylsulfonyl fluoride). Extracts were incubated at 4°C for 1 h, then centrifuged for 30 min at maximum speed. Supernatants were collected for further analysis. To obtain the nuclear‐enriched fraction, samples were collected in the same quantity (0.2 g of 12‐d‐old fresh leaves), and the nuclear proteins were extracted as described previously (Kaufmann *et al*., 2010).

For the CLD1‐GFP accumulation assay, to obtain total proteins, rice protoplasts were collected and treated with lysis buffer (cat. no. B7345; Sigma‐Aldrich). After incubation at 4°C for 1 h, the extracts were centrifuged for 30 min at maximum speed. Supernatants were collected for further analysis. The nuclear proteins of the rice protoplasts were extracted using a Minute Cytoplasmic and Nuclear Kit (Invent Biotechnologies, Eden Prairie, MN, USA) according to the manufacturer's instructions.

### Protein degradation assay

The recombinant His‐CLD1 was synthesized using the TNT SP6 High‐Yield Wheat Germ Protein Expression System (Promega) according to the manufacturer’s instructions. The equivalent amount of His‐CLD1 was added to 120 μl crude total proteins (20 µg µl^–1^) extracted from indicated plants (non‐KO1^*OsCUC1*^ or *oscuc1*‐KO1) using reaction buffer (50 mM Tris‐HCl, pH 7.8, 100 mM NaCl, 0.1% (v/v) Tween 20, 10% (v/v) glycerol and 20 mM β‐mercaptoethanol). The mixture was incubated at 25°C and sampled at indicated time points (0, 3, 6 and 9 h). The abundance of remaining His‐CLD1 in these samples was detected by immunoblotting using an anti‐His (TransGen) antibody. The original blot images are shown in Fig. S1.

### Statistical analysis

Statistical analysis was performed by Student's *t*‐test. *P*‐values of < 0.05 were considered to indicate statistical significance. *P*‐values of < 0.01 were considered to indicate statistically high significance. Statistical calculations were performed using Microsoft Excel 2010.

## Results

### Knocking out *OsCUC1* and *OsCUC3* in rice leads to defects in meristem/organ boundary specification

To identify the *OsCUC* genes, we searched for homologs of *CUC1*, *CUC2* and *CUC3* in rice. Sequence alignment indicated that *LOC_Os06g23650* (*Os06g0344900*) is the closest homolog of both *CUC1* and *CUC2*, hereafter referred to as *OsCUC1*; *LOC_Os08g40030* (*Os08g0511200*) shares the greatest similarity with *CUC3*, and we therefore named it *OsCUC3*. Both genes were previously uncharacterized. *OsCUC1* encodes a 373 amino acid (aa) protein with a typical NAC domain; while *OsCUC3* encodes a 340‐aa NAC protein (Fig. S2a). Both *OsCUC1* and *OsCUC3* belong to the NAM subgroup of the NAC gene family (Fang *et al*., 2008; Nuruzzaman *et al*., 2010). A phylogenetic tree analysis showed that OsCUC1 is the homologue of Arabidopsis CUC1, and it is also very similar to Arabidopsis CUC2, petunia NAM, tomato GOBLET, strawberry FveCUC2a, FveCUC2b, and FveCUC2c. OsCUC3 is the homologue of CUC3 in Arabidopsis and FvH4_5g12090 in strawberry (Fig. S2c).

**Fig. 1:**
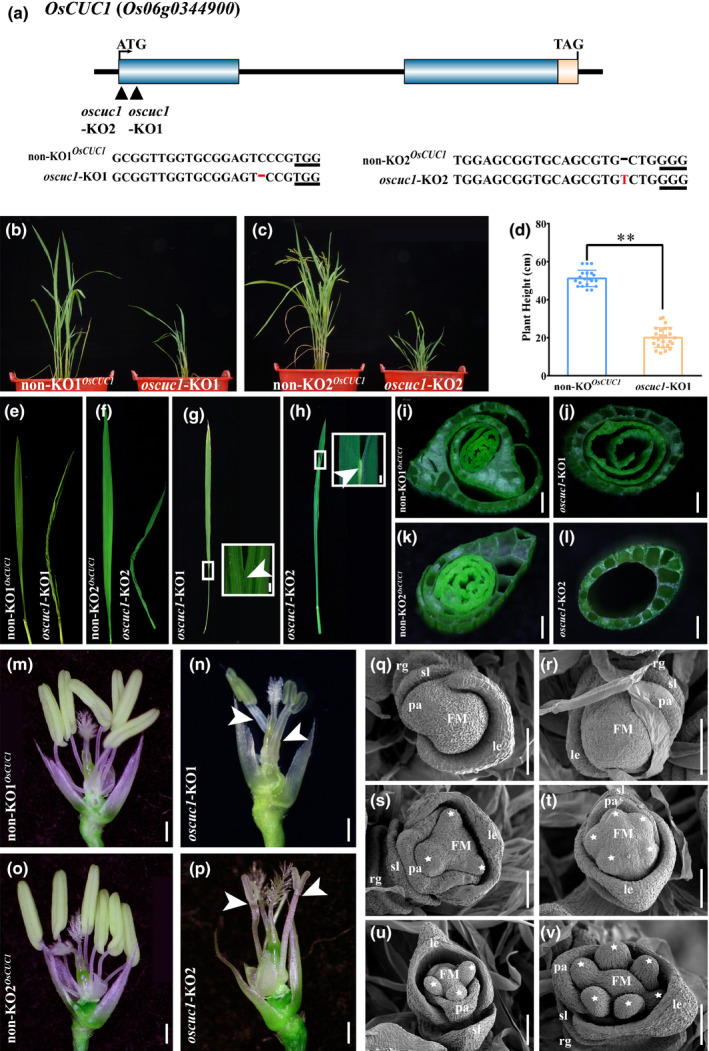
Appearance of rice *oscuc1* knockout mutants. (a) Schematic diagram of *OsCUC1* gene structure and the CRISPR/Cas9 target sites. The black triangles indicate the two individual target sites for knockout of *OsCUC1*. The PAM site is underlined. The mutation site is shown in red. (b, c) The whole plant phenotypes of *oscuc1‐*KO1 and *oscuc1‐*KO2. (d) The height of mature non‐KO1*^OsCUC1^* and *oscuc1‐*KO1 plants. Values are shown as means ± SD (20 non‐KO1*^OsCUC1^* plants and 25 *oscuc1‐*KO1 were used for the statistical analysis). **, *P* < 0.01 (Student's *t*‐test). (e, f) The twisted‐rolling leaves in *oscuc1‐*KO1 and *oscuc1‐*KO2. (g, h) Fusion of leaf blade in *oscuc1‐*KO1 and *oscuc1‐*KO2. The fusion regions outlined by small white rectangles are shown in detail on the right‐hand side of their respective panels. The arrows indicate the fusion of the leaf blades. (i–l) Cross‐section of the leaf sheath of *oscuc1‐*KO1, *oscuc1‐*KO2 and their non‐KO control. (m–p) Knocking out *OsCUC1* leads to fusion in filaments and reduction in stamens. The arrows indicate the fusion of the filaments. (q–v) Scanning electron micrographs of early‐arising non‐KO1*^OsCUC1^* and *oscuc1*‐KO1 floret. FM, flora meristem; le, lemma; pa, palea; sl, sterile lemma; rg, rudimentary glumes. The white asterisks represent the primordia of the stamens. Bars: (g–p) 500 μm; (q–v) 100 μm.

**Fig. 2:**
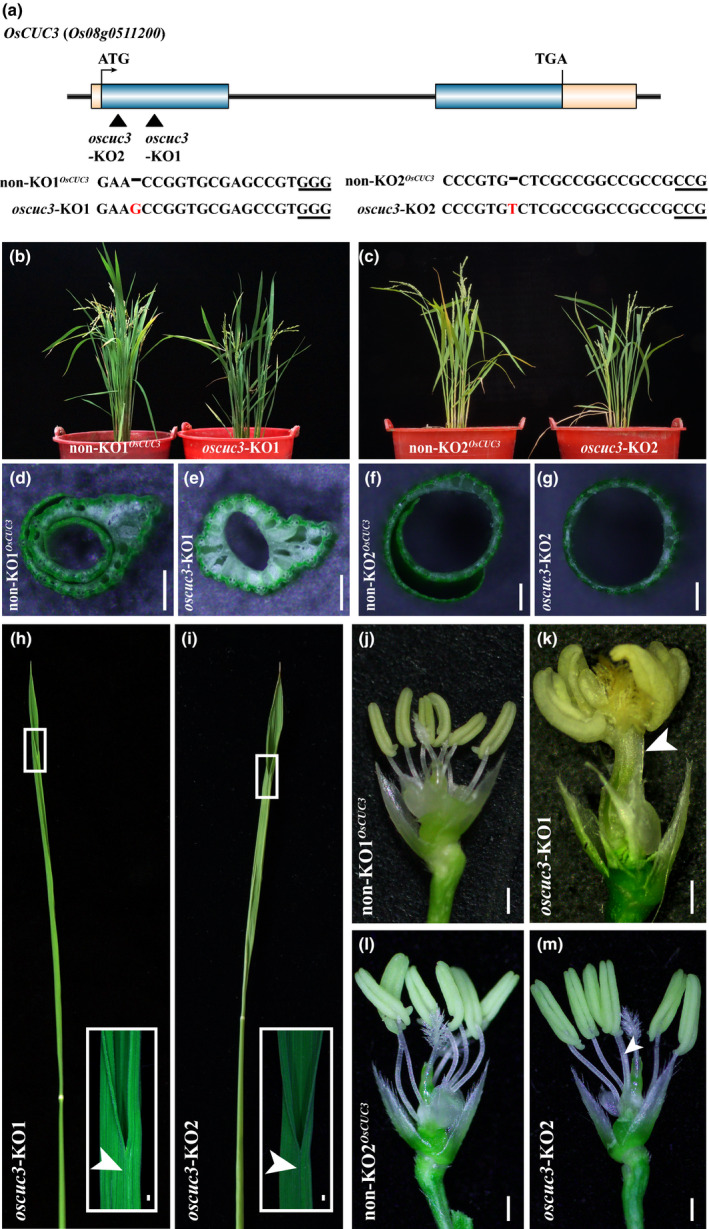
Appearance of rice *oscuc3* knockout mutants. (a) Schematic diagram of *OsCUC3* gene structure and the CRISPR/Cas9 target sites. The black triangles indicate the two individual target sites for knockout of *OsCUC3*. The PAM site is underlined. The mutation site is shown in red. (b, c) The whole plant phenotype of *oscuc3‐*KO1 and *oscuc3‐*KO2. (d–g) Cross‐section of the leaf sheath of *oscuc3‐*KO1, *oscuc3‐*KO2 and their non‐KO control. (h, i) Fusion of leaf blade in *oscuc3‐*KO1 and *oscuc3‐*KO2. The fusion region outlined by the small white rectangle is shown in detail in the bottom right corner. The arrows indicate the fusion of the leaf blades. (j–m) Knocking out *OsCUC3* leads to fusion in filaments. The arrow indicates the fusion of two filaments of *oscuc3‐*KO2. Bars, 500 μm.

To understand the biological function of *OsCUC1* and *OsCUC3*, we employed CRISPR/Cas9 to produce two individual knockout lines for each of these two genes (Figs 1a, 2a). For *OsCUC1*, a 1 bp deletion for *oscuc1*‐KO1 and a 1 bp insertion for *oscuc1*‐KO2 (at different sites) lead to a frame‐shift with premature transcription termination (Fig. S3a), while both *oscuc3*‐KO1 and *oscuc3*‐KO2 harbor a 1 bp insertion at different sites of the coding region resulting in a premature stop codon (Fig. S3b).

In comparison with the non‐KO control line, which harbored the intact *OsCUC1,* both homozygous *oscuc1*‐KO lines showed multiple defects in rice development, including the fusion of leaves and filaments, decreased stamen numbers, reduced plant height and twisted‐rolling leaves (Fig. 1b–p; Table S3). During the vegetative growth stage, fusion could be observed in the leaf blade (Figs 1g,h, S4a,b) as well as in the whole leaf sheath (Figs 1i–l, S4c,d) of the *oscuc1*‐KO plants. The tube‐like leaf sheath physically prevented the outgrowth of the new leaf or the panicle. During the reproductive growth stage, we also observed defects in floral development in *oscuc1*‐KO, in which the florets displayed fusions of two or more filaments (Fig. 1m–p). This fusion in the mutants indicated the function of *OsCUC1* in organ boundary specification. At the same time, most of the florets showed reduced stamen numbers (Fig. 1m–p; Table S3). An SEM assay showed that not all of the stamen primordia correctly formed in the early floral developmental stage in the *oscuc1*‐KO plants, indicating that *OsCUC1* also functions in meristem boundary specification (Fig. 1q–v). We did not obtain any seeds from any of the individual T_0_ homozygous *oscuc1*‐KO plants, while the non‐KO plants displayed normal fertility. This result may be attributable to the development of aberrant stamens, which may lead to pollen defects in the mutant plants. The pollen viability test indicated that almost no pollen was produced in the KO plants (Fig. S5). Furthermore, pollen from wild‐type plants (Nipponbare) was used to pollinate the gynoecia of *oscuc1*‐KO1. In contrast with the self‐pollinated florets, the cross‐pollinated florets maintained normal development in later stages and yielded fertile seeds (Fig. S6a–e). We further investigated whether these seeds could develop normally. No defects were detected in any developmental stage in these F_1_ progeny (*oscuc1*‐KO1/+). In the F_2_ population, the homozygous *oscuc1*‐KO1 plants showed the same phenotype as the T_0_ transgenic plants, while the non‐KO plants and heterozygous mutants developed normally (Fig. S6f,g).

Both of the *oscuc3‐*KO lines exhibited defects in meristem/organ boundary specification in the vegetative growth stage as well as in the reproductive growth stage, including the fusion of leaves and filaments, and defects in stamen identification (Fig. 2b–m; Table S3), which were highly similar to those of the *oscuc1‐*KO lines. However, knocking out *OsCUC3* did not lead to dwarf plant stature or twisted‐rolling leaves. In addition, in contrast to the infertility of the *oscuc1*‐KO plants, fertile seeds could be obtained from both of the *oscuc3*‐KO lines. Similar to the *oscuc1*‐KO1/+ plants, the heterozygous *OsCUC3* mutant plants showed no defects during any developmental stages (Fig. S6h,i).

### The expression profiles of *OsCUC1* and *OsCUC3*

**Fig. 3:**
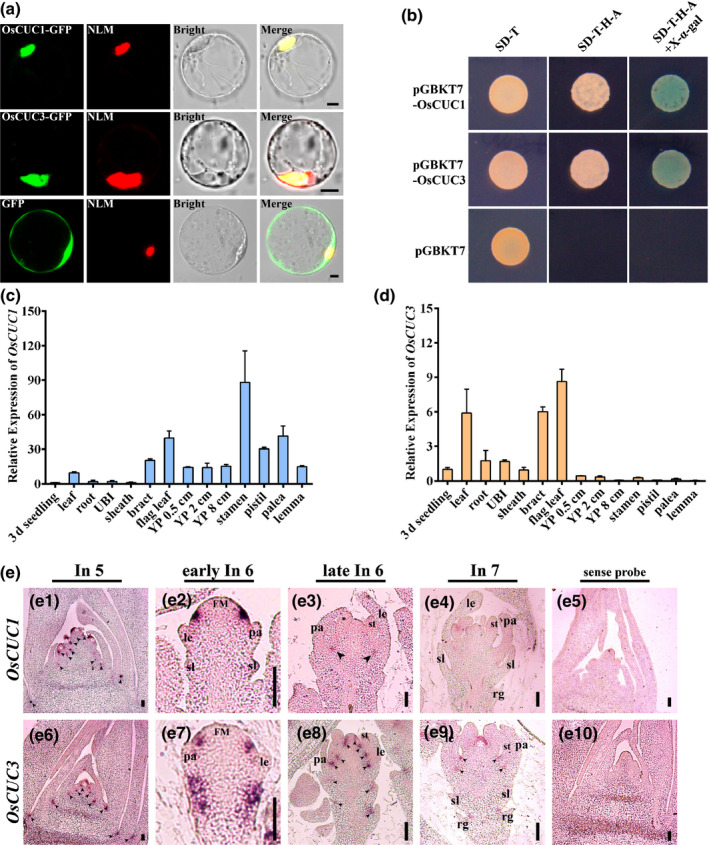
The expression patterns of *OsCUC1* and *OsCUC3* in rice. (a) Subcellular localization of OsCUC1 and OsCUC3. NLM, nuclear localization marker (Ghd7‐mCherry). Bars, 5 μm. (b) Transcriptional activity analysis of OsCUC1 and OsCUC3. The empty vector *pGBKT7* was used as a negative control. (c, d) Relative expression of *OsCUC1* and *OsCUC3* in seedling (3 d after germination), leaf, root, UBI (unelongated basal internode), leaf sheath, bract, flag leaf, YP 0.5 cm (young panicle 0.5 cm), YP 2 cm, YP 8 cm, stamen, pistil, palea and lemma. Values are shown as means ± SD (*n* = 3). (e) *In situ* hybridization of *OsCUC1* and *OsCUC3*. (e1–e4) Accumulation of *OsCUC1* in wild‐type plants. (e6–e9) Accumulation of *OsCUC3* in wild‐type plants. (e5, e10) Negative controls with sense probes. The black arrows indicate the accumulation regions of the mRNA. FM, flora meristem; le, lemma; pa, palea; rg, rudimentary glumes; sl, sterile lemma; st, stamens. Bars, 50 μm.

NAC family proteins have been shown to function as transcription factors (Xie *et al*., 1999; Vroemen *et al*., 2003). Since *OsCUC1* and *OsCUC3* encode NAC domain proteins, we used GFP fluorescence to determine their subcellular localization by transiently expressing OsCUC1‐GFP or OsCUC3‐GFP in‐frame fusion proteins in rice protoplasts. The results revealed that both OsCUC1 and OsCUC3 are localized to the nucleus (Fig. 3a). In addition, to determine whether these genes have transactivation activity, OsCUC1 and OsCUC3 were fused to the GAL4 DNA binding domain and transformed into the yeast strain AH109. The yeast strains containing *pBD‐OsCUC1* or *pBD‐OsCUC3* grew in Trp‐, His‐ and Ade‐deficient SD medium while the negative control did not grow in triple‐deficient SD medium (Fig. 3b), which indicated that both genes have transactivation activity. Taken together, these data indicated that OsCUC1 and OsCUC3 function as transcription factors.

We used qRT‐PCR to study the expression patterns of *OsCUC1* and *OsCUC3* throughout plant development. *OsCUC1* was highly expressed in the leaf, bract, flag leaf, and reproductive organs, especially in the stamens (Fig. 3c). In comparison to that of *OsCUC1*, the expression level of *OsCUC3* was lower in all tested organs. However, *OsCUC3* transcripts accumulated more abundantly in the vegetative organs than in the reproductive organs (Fig. 3d). To obtain the details of the expression patterns of these two genes, we carried out an *in‐situ* hybridization assay (Fig. 3e). In the early floral developmental stage (In 5), *OsCUC1* and *OsCUC3* exhibited highly overlapping expression patterns, with both mRNAs accumulated mainly in the boundaries (between the leaves and between the meristems) as well as within the meristems (Fig. 3e1,e6). In early In 6 stage, *OsCUC1* mRNA was strongly and specifically accumulated at the region where the stamen primordia will arise (Fig. 3e2). Afterward, the expression of *OsCUC1* became diffused and uniform in the flower organs (Fig. 3e3,e4). For *OsCUC3*, in early In 6 stage, its mRNA was detected in the same region where *OsCUC1* mRNA accumulated (Fig. 3e7); in addition, *OsCUC3* was also detected in the primordia of sterile lemmas and rudimentary glumes (Fig. 3e7). In the later stages (late In 6 and In 7), in contrast to *OsCUC1*, *OsCUC3* was mainly detected in the boundaries between the flower organs (Fig. 3e8,e9).

### OsCUC1 and OsCUC3 dimerize and function in a partially redundant manner in boundary specification and SAM activity maintenance

**Fig. 4:**
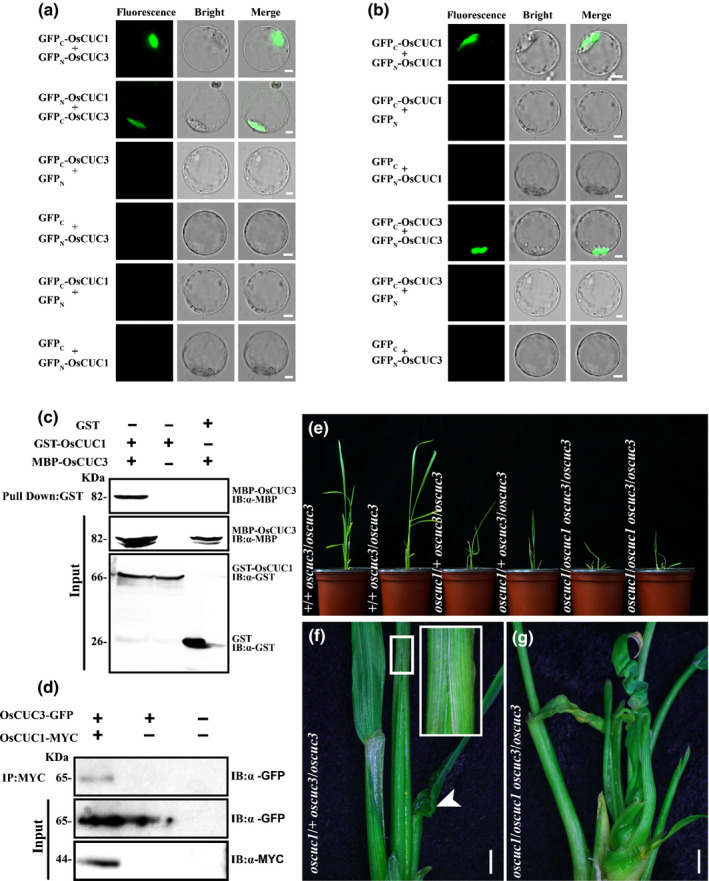
OsCUC1 and OsCUC3 dimerize and function redundantly in rice. (a, b) BiFC assays showing the heterodimerization and homodimerization of OsCUC1 and OsCUC3. Co‐expression of GFP_C_‐OsCUC3 plus GFP_N_, GFP_C_ plus GFP_N_‐OsCUC3, GFP_C_‐OsCUC1 plus GFP_N_, and GFP_C_ plus GFP_N_‐OsCUC1 were used as negative controls. Bars, 5 μm. (c) Interaction between OsCUC1 and OsCUC3, analysed by *in vitro* pull‐down assay. Recombinant GST‐OsCUC1 and MBP‐OsCUC3 proteins were used for the pull‐down assay. IB, immunoblot. (d) Interaction between OsCUC1 and OsCUC3, analysed by *in vivo* CoIP assay. After the co‐transformation of OsCUC1‐MYC and OsCUC3‐GFP in rice protoplasts, total proteins of protoplasts were immunoprecipitated using an anti‐MYC antibody and were detected with anti‐GFP and anti‐MYC antibodies. IB, Immunoblot; IP, immunoprecipitation. (e) The phenotypes of +/+ *oscuc3*/*oscuc3*, *oscuc1*/+ *oscuc3*/*oscuc3* and *oscuc1*/*oscuc1 oscuc3*/*oscuc3*. (f) The fusion of leaf sheath and twisted‐rolling leaf in *oscuc1*/+ *oscuc3*/*oscuc3*. The fusion region of the leaf sheath outlined by a small white rectangle in the main image is shown in detail in the top right corner; the arrow indicates the twisted‐rolling leaf. Bar, 500 μm. (g) The enhanced defects in the *oscuc1*/*oscuc1 oscuc3*/*oscuc3* double mutant. Bar, 500 μm.

To understand the relationship between OsCUC1 and OsCUC3, we first studied the protein–protein interaction of these two proteins. A bimolecular fluorescence complementation (BiFC) assay showed that OsCUC1 and OsCUC3 could form homodimers or heterodimers (Fig. 4a,b). An *in vivo* co‐immunoprecipitation (Co‐IP) assay and an *in vitro* pull‐down assay further confirmed the dimerization between OsCUC1 and OsCUC3 (Fig. 4c,d). In Arabidopsis, due to the *miR164* function in negatively regulating *CUC1* and *CUC2*, the interaction between the CUC proteins cannot be detected in protoplasts by BiFC. However, we detected that the *miR164*‐resistant versions of CUC1 and CUC2 (mCUC1 and mCUC2) formed homodimers, while mCUC1‐mCUC2, mCUC1‐CUC3 and mCUC2‐CUC3 formed heterodimers (Fig. S7). These results were largely consistent with previous findings (Rubio‐Somoza *et al*., 2014; Gonçalves *et al*., 2015), indicating the conserved protein–protein interaction behavior of CUC proteins in both rice and Arabidopsis.

Furthermore, we developed double mutant plants for these two genes. In contrast with the single mutant plants, which showed no defects during the early seedling stage (Fig. S6f, h), the *oscuc1*/+ *oscuc3*/*oscuc3* plants exhibited leaf sheath fusion and twisted‐rolling leaves; these defects were greatly enhanced in the *oscuc1*/*oscuc1 oscuc3*/*oscuc3* plants (Fig. 4e–g). Although loss of function of *OsCUC1* and *OsCUC3* did not lead to defects in SAM initiation (Fig. S8a–d), the maintenance of SAM activity was markedly affected – the development of the double mutant plants was arrested in the early seedling stage, and they eventually died soon after that (Fig. S8e–l). Together, these results indicate that OsCUC1 and OsCUC3 dimerize and function in a partially redundant manner in boundary specification and SAM activity maintenance.

### *osa‐miR164c* is involved in organ boundary specification and leaf development by negatively regulating *OsCUC1*

**Fig. 5:**
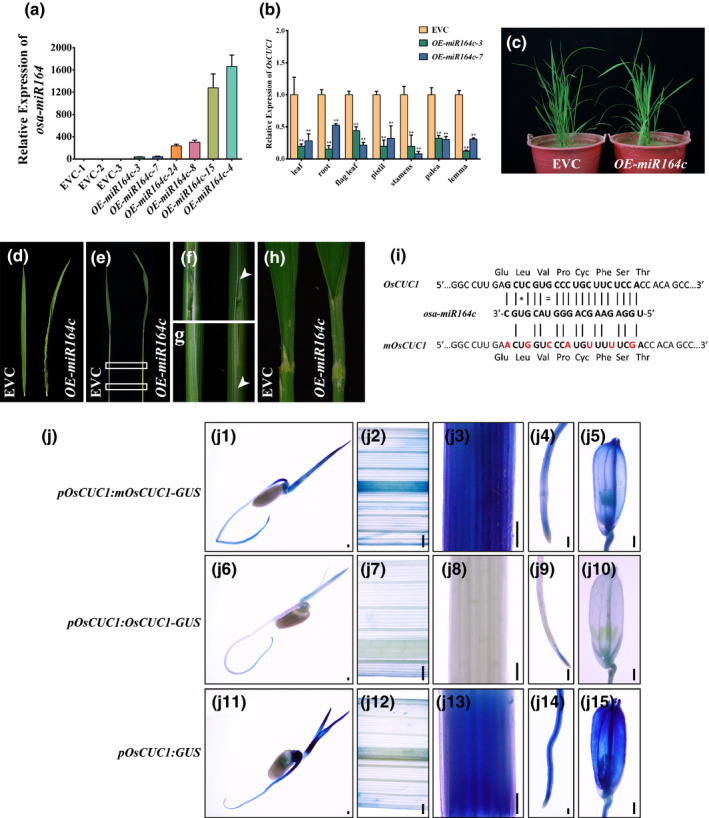
*osa‐miR164c* is involved in boundary specification and leaf development by negatively regulating *OsCUC1* in rice. (a) Relative expression of *osa‐miR164c* in different individual *OE‐osa‐miR164c* lines and empty‐vector control lines (EVC). Values are shown as means ± SD (*n* = 3). (b) Relative expression of *OsCUC1* in EVC and two *OE‐osa‐miR164c* lines in different tissues. Values are shown as means ± SD (*n* = 3). **, *P* < 0.01 (Student's *t*‐test). (c) The phenotype of *OE‐osa‐miR164c* and EVC lines. (d) The twisted‐rolling leaf in *OE‐osa‐miR164c*. (e) Overexpressing *osa‐miR164c* leads to fusion in leaf sheaths. (f, g) Detailed images of the fusion regions depicted in (e); the arrows indicate the fusion regions on the leaf sheath. (h) Overexpressing *osa‐miR164c* leads to fusion in the leaf blade. (i) Schematic diagram depicting the construction of *mOsCUC1*. The red letters indicate mutant nucleotides introduced into the *osa‐miR164* target site in the *mOsCUC1‐GUS* transgene, which interrupts the *osa‐miR164* target site without changing the amino acid residues. Watson–Crick base pairing between the mRNA and *osa‐miR164c* is indicated by black lines. Mismatches and G:U wobbles are indicated by a star or equals sign, respectively. (j) Gus staining for *pOsCUC1::mOsCUC1‐GUS*, *pOsCUC1::OsCUC1‐GUS* and *pOsCUC1::GUS* in seedling (j1, j6, j11), leaf blade (j2, j7, j12), leaf sheath (j3, j8, j13), root (j4, j9, j14), and floret (j5, j10, j15). Bars, 200 μm.

In Arabidopsis, it has been demonstrated that *miR164* negatively regulates *CUC1* and *CUC2* (Baker *et al*., 2005; Nikovics *et al*., 2006; Sieber *et al*., 2007; Raman *et al*., 2008). To test whether *OsCUC1* is regulated by microRNA, we searched for the corresponding microRNA using *OsCUC1* as a putative target in the Plant Non‐coding RNA Database, (PNRD http://structuralbiology.cau.edu.cn/PNRD/index.php) (Yi *et al*., 2014). The results indicated that *OsCUC1* may be regulated by all members of the rice microRNA *osa‐miR164* family (*osa‐miR164a* to *osa‐miR164f*). These findings implied that *osa‐miR164* may also be involved in organ boundary specification and leaf development by negatively regulating *OsCUC1*. The bioinformatics analysis by PNRD indicated that *OsCUC1* is considered to be the target of *osa‐miR164c* with a high expectation value; moreover, only four genes, including *OsCUC1*, are predicted to be the potential targets of *osa‐miR164c*, while the other *osa‐miR164* targets more genes. Thus, *osa‐miR164c* was selected for further study. The results from RT‐PCR followed by Sanger sequencing indicated that the real existence of the *osa‐miR164c* is supported by transcript evidence (Fig. S9a). Moreover, the qRT‐PCR results indicated that *osa‐miR164c* was accumulated highly in leaf tissues, but almost no expression could be detected in the mature flower organs, in which *OsCUC1* is relatively highly expressed (Fig. S9b). To verify the functions of *osa‐miR164c* in regulating boundary specification and leaf morphology, we overexpressed *osa‐miR164c* in the genetic background of Nipponbare. In comparison to the control plants carrying the empty vector, *OsCUC1* was significantly downregulated in both the vegetative and reproductive organs of *OE‐osa‐miR164c* plants (Fig. 5a–c). Similar to *oscuc1*‐KO, the *OE‐osa‐miR164c* plants also showed defects in organ boundary separation and leaf development (Fig. 5d–h). However, unlike in *oscuc1*‐KO, the stamens developed normally in *OE‐osa‐miR164c*, indicating that a low expression level of *OsCUC1* is sufficient for maintaining normal organ separation in the reproductive growth stage but not in the vegetative growth stage. Notably, two transgenic lines with higher expression of *osa‐miR164c* (*OE‐osa‐miR164c*‐4 and *OE‐osa‐miR164c*‐15) showed more severe defects than the others, and no seeds could be obtained from these two lines.

*OsCUC1* contains an *osa‐miR164*‐target sequence in the second exon. To further test the effects of *osa‐miR164* on *OsCUC1*, we generated transgenic lines carrying an *OsCUC1* in‐frame fusion with the *β‐glucuronidase* (*GUS*) gene driven by its endogenous promoter with or without synonymous mutations in the *osa‐miR164*‐targeted site (*pOsCUC1::OsCUC1‐GUS* and *pOsCUC1::mOsCUC1‐GUS*) (Fig. 5i). The *pOsCUC1::mOsCUC1‐GUS* lines showed a relatively high level of GUS staining in most of the organs (Fig. 5j1–j5), while only minor staining was observed in the *pOsCUC1::OsCUC1‐GUS* lines (Fig. 5j6–j10). These results strongly suggest that *osa‐miR164c* functions by dampening the transcription level of *OsCUC1*. In agreement with this idea, the transcriptional reporter of *OsCUC1* (*pOsCUC1::GUS*) showed a strong expression pattern, as expected (Fig. 5j11–j15).

### *osa‐miR164* targeting *OMTN4* and *OMTN6* may not be involved in meristem/organ boundary specification or leaf development

In addition to *OsCUC1*, five other NAC (OMTN) genes, *OMTN1* (*ONAC027*), *OMTN2* (*ONAC004*/*OsNAC2*), *OMTN3* (*ONAC060*), *OMTN4* (*ONAC011*) and *OMTN6* (*ONAC104*), are considered to be putative targets of *osa‐miR164* (Fang *et al*., 2014). We have shown that *osa‐miR164c* plays a critical role in boundary specification and leaf development by downregulating *OsCUC1*. Our qRT‐PCR results indicated that only *OsCUC1* – and not the other five putative *osa‐miR164* targets – was significantly downregulated in all the organs tested in *OE‐osa‐miR164c* plants (Figs 5b, S10). In a previous study, OMTN4‐RNAi (RNA interference) and OMTN6‐RNAi transgenic plants were reported to show severe abnormal phenotypes, such as twisted‐rolling leaves and fused organs, which were similar to those of the *oscuc1*‐KO plants (Fang *et al*., 2014). To verify whether *OMTN4* and *OMTN6* function in controlling organ specification and leaf development, we knocked out these two genes using CRISPR/Cas9 in the Nipponbare genetic background. We did not observe any fused or twisted‐rolling leaves for the homozygous knockout (KO) mutants of these two genes during the vegetative growth stage, and in the reproductive stage, defects in the florets were barely detected. Moreover, no significant defects could be observed from two other mutant lines with the Zhonghua11 background (*omtn4* ^ZH11^ and *omtn6* ^ZH11^) either (Fig. S11; Table S4). Therefore, the defects in the two RNAi lines may be caused by RNAi off‐target effects. Thus, we postulated that the targets of *osa‐miR164*, *OMTN4* and *OMTN6* may not respond to meristem/organ boundary specification or leaf development as *OsCUC1* does.

### OsCUC1 physically interacts with CLD1 and maintains CLD1 stability in the nucleus to control leaf morphology

**Fig. 6:**
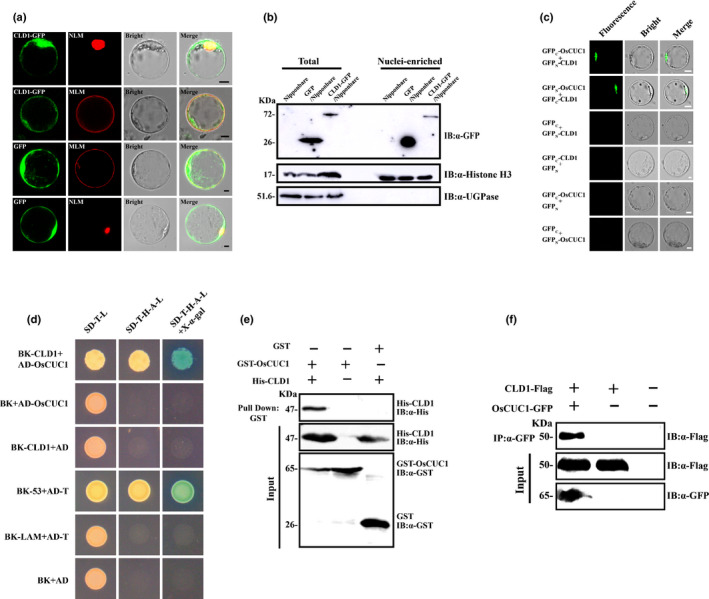
OsCUC1 interacts with CLD1 in rice. (a) CLD1 is localized to the cell membrane as well as the nucleus. Upper row: co‐localization of CLD1‐GFP and an mCherry‐tag nuclear localization marker. Upper middle row: co‐localization of CLD1‐GFP and an mCherry‐tag membrane localization marker. Lower middle row: co‐localization of green fluorescent protein (GFP) and an mCherry‐tag membrane localization marker as a control. Lower row: co‐localization of GFP and an mCherry‐tag nuclear localization marker as a control. MLM, membrane localization marker (OsRac3‐mCherry); NLM, nuclear localization marker (Ghd7‐mCherry). Bars, 5 μm. (b) Accumulation of CLD1‐GFP in the nucleus. Rice (Nipponbare) protoplast transiently expressed GFP or CLD‐GFP. CLD1‐GFP proteins were detected using anti‐GFP antibodies in total proteins of the rice protoplast or in the nuclei‐enriched fraction only. Histone H3 served as a nuclear marker and UGPase (UDP‐glucose pyrophosphorylase) as a cytoplasmic marker. (c) BiFC assay. Co‐expression of GFP_C_ plus GFP_N_‐CLD1, GFP_C_‐CLD1 plus GFP_N_, GFP_C_‐OsCUC1 plus GFP_N_, and GFP_C_ plus GFP_N_‐OsCUC1 were used as negative controls. Bars, 5 μm. (d) Y2H assay. The combination of BK‐53 plus AD‐T was used as a positive control, while BK‐CLD1 plus AD, BK‐LAM plus AD‐T, and BK plus AD were used as negative controls. (e) *In vivo* co‐immunoprecipitation (CoIP) assay. After the co‐transformation of OsCUC1‐GFP and CLD1‐Flag in rice protoplasts, total proteins of protoplasts were immunoprecipitated using an anti‐GFP antibody and were detected with anti‐GFP and anti‐Flag antibodies. IB, immunoblot; IP, immunoprecipitation. (f) *In vitro* pull‐down assay. Recombinant GST‐OsCUC1 and His‐CLD1 proteins were used for the pull‐down assay. IB, immunoblot.

**Fig. 7:**
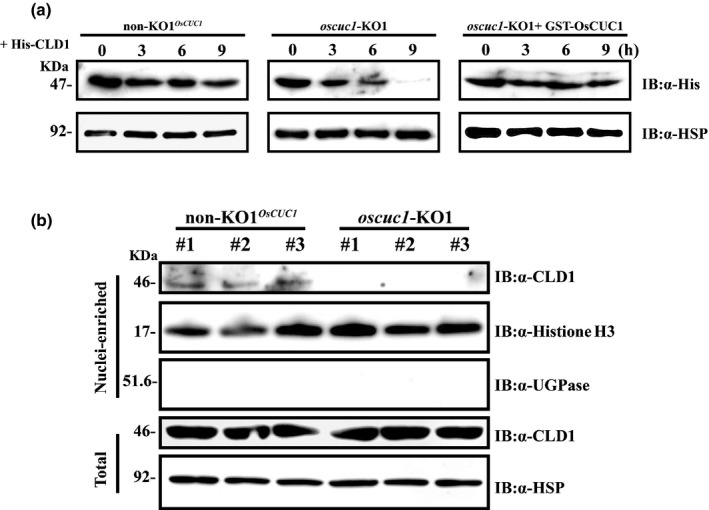
OsCUC1 stabilizes CLD1 in the nucleus in rice. (a) *In vitro* protein degradation assay. Recombinant His‐CLD1 protein was incubated with total crude extract from non‐KO1*^OsCUC1^* (left) and *oscuc1*‐KO1 (middle, without GST‐OsCUC1; right, with GST‐OsCUC1). HSP (Hsp90) was used as a loading control. (b) The accumulation of CLD1 in the nucleus depends on OsCUC1 functioning; accumulation of CLD1 in non‐KO1*^OsCUC1^* and *oscuc1*‐KO1 plants is shown. CLD1 was detected with an anti‐CLD1 antibody. Histone H3 served as a nuclear marker, and UGPase (UDP‐glucose pyrophosphorylase) served as a cytoplasmic marker. HSP (Hsp90) was used as a loading control. Three independent plants of each line were used for the assay.

Knocking out *OsCUC1* in rice gives rise to defects in leaf development, which resemble the phenotype of the *curled leaf and dwarf 1* (*cld1*) mutants (Li *et al*., 2017). However, the expression level of *CLD1* showed no significant change in the KO plants (Fig. S12). Thus, we posited that OsCUC1 might interact with CLD1 and function in maintaining the stability of the CLD1 protein. In previous research, it has been demonstrated that *CLD1*, also known as *Semi‐Rolled Leaf 1* (*SRL1*), encodes a glycophosphatidylinositol (GPI)‐anchored membrane protein, which localizes predominantly at the plasma membrane and modulates leaf development (Xiang *et al*., 2012; Li *et al*., 2017). To verify whether CLD1 can interact with OsCUC1, we first tested whether CLD1 is located in the nucleus, as OsCUC1 is. We transiently co‐expressed CLD1‐GFP in‐frame fusion proteins with a nuclear localization marker or membrane localization marker in rice protoplasts. Green fluorescence protein fluorescence indicated that CLD1 was located in both the membrane and the nucleus (Fig. 6a). Moreover, CLD1‐GFP was detected in the nuclei‐enriched fraction by immunoblotting (Fig. 6b). This result further confirmed the nuclear localization of CLD1. We then studied the protein–protein interactions between CLD1 and the NAC proteins; BiFC, yeast‐two‐hybrid (Y2H), *in vivo* Co‐IP and *in vitro* pull‐down assays revealed the interactions between CLD1 and OsCUC1 (Fig. 6c–f). By contrast, we did not detect any interactions between CLD1 and OsCUC3 or two other NAC proteins (the putative *osa‐miR164* targets OMTN4 and OMTN6) by either BiFC or Y2H assays (Fig. S13). In the *in vitro* assay conditions, recombinant His‐CLD1 was stable in the crude protein extract from non‐KO1*^OsCUC1^*. By contrast, His‐CLD1 proteins were rapidly degraded after the addition of crude protein extract from *oscuc1*‐KO1, while the degradation of His‐CLD1 was greatly slowed down when recombinant GST‐OsCUC1 was first mixed with His‐CLD1 (Fig. 7a). Moreover, we carried out an assay for CLD1 stability *in planta*. Immunoblotting assays revealed that CLD1 was not detected in the nucleus‐enriched fraction from *oscuc1‐*KO plants, indicating that the nuclear accumulation of CLD1 is dependent on OsCUC1 functioning (Fig. 7b). These results further confirmed the role of OsCUC1 in the stabilization of CLD1. Overall, the protein–protein interaction between OsCUC1 and CLD1 may stabilize CLD1 in the nucleus; loss of function of OsCUC1 may lead to acceleration of CLD1 degradation in the nucleus, resulting in a phenotype with twisted‐rolling leaves, which is similar to that of the *cld1* mutant.

## Discussion

In plants, members of CUP‐SHAPED COTYLEDON (CUC)⁄NO APICAL MERISTEM (NAM), a group of plant‐specific NAC transcription factors, play critical roles in boundary specification, including CUC1, 2 and 3 in Arabidopsis, CUPULIFORMIS (CUP) in *Antirrhinum majus*, GOBLET (GOB) in tomato (*Solanum lycopersicum*), NO APICAL MERISTEM (NAM) in *Petunia* and MtNAM in *Medicago truncatula* (Souer *et al*., 1996; Aida *et al*., 1997; Takada *et al*., 2001; Vroemen *et al*., 2003; Weir *et al*., 2004; Berger *et al*., 2009; Cheng *et al*., 2012). On the other hand, *miR164* is involved in multiple developmental processes by negatively regulating its targets, which are always NAC transcription factors. In Arabidopsis*, miR164* regulates boundary specification, leaf margin serration, lateral root development, pathogen‐induced cell death, age‐dependent cell death and the salt stress response by targeting different NAC genes (Guo *et al*., 2005; He *et al*., 2005; Nikovics *et al*., 2006; Sieber *et al*., 2007; Raman *et al*., 2008; Kim *et al*., 2009; Lee *et al*., 2017); in kiwifruit (*Actinidia spp*.), an interplay between ade‐*miR164* and *AdNAC6/7* regulates fruit ripening (Wang *et al*., 2019); in strawberry (*Fragaria vesca*), the *miR164*‐*CUC2* module regulates leaf and flower development (Zheng *et al*., 2019); in *M. truncatula*, *miR164*‐*MtNAC1* pathway is involved in root and symbiotic nodule development (D'haeseleer *et al*., 2011); and in wheat (*Triticum aestivum*), *TaNAC21/22*, the targets of tae‐*miR164*, play an important role in regulating the resistance of host plants to stripe rust (Feng *et al*., 2014). In rice, overexpression of *osa‐miR164b* led to dwarf plant architecture and small panicles (Jiang *et al*., 2018). Transgenic plants overexpressing the *osa‐miR164*‐targeted genes, including *OMTN2* (*ONAC004*/*OsNAC2*), *OMTN3* (*ONAC060*), *OMTN4* (*ONAC011*) and *OMTN6* (*ONAC104*) increased the sensitivity to drought stress at the reproductive growth stage, indicating that these genes appear to be associated with the response to abiotic stresses (Fang *et al*., 2014).

### The role of *OsCUC1* and *OsCUC3* in SAM formation and maintenance

In dicots, it has been demonstrated that *NAM* in *Petunia*, *MtNAM* in *Medicago*, *CUP* in *Antirrhinum* and *CUCs* in Arabidopsis are involved in SAM formation. Mutations in *NAM*, *MtNAM* and *CUP* lead to fusion in cotyledons, and no apical meristem can be formed. In *Medicago*, the development of the strong mutant of *MtNAM* is arrested in the fused cotyledon stage (Cheng *et al*., 2012). By contrast, in *Petunia*, the *nam* mutant occasionally produces escape shoots, which develop normally in the vegetative stage, but show defects in flower development (Souer *et al*., 1996). In *Antirrhinum*, the *cup* mutant always produces escape shoots; however, these escape shoots display defects during all developmental stages (Weir *et al*., 2004). In Arabidopsis, *CUC1* and *CUC2* function redundantly in SAM formation, and seedlings of the *cuc1 cuc2* double mutant, which completely lacks a SAM, exhibit a cup‐shaped cotyledon (Aida *et al*., 1997). Further study indicated that *cuc2 cuc3* double mutant seedlings, but not *cuc1 cuc3* seedlings, have no functional SAM. Thus, only *cuc2* in combination with *cuc1* and/or *cuc3* leads to the absence of the SAM (Vroemen *et al*., 2003).

In our study, we found that the *oscuc1 oscuc3* double mutant produces aberrant organs at the seedling stage, and the development of the double mutant seedling is arrested. These results indicated that, in addition to *OsCUC1* and *OsCUC3*, other factor(s) may be involved in the regulation of SAM initiation, and loss of function of these two genes alone is not sufficient to prevent SAM formation. However, *OsCUC1* and *OsCUC3* are critical for maintaining normal SAM activity. Plants without functional *OsCUC1* and *OsCUC3* products stop developing and ultimately die at the seedling stage.

### Functional divergence between OsCUC1 and OsCUC3

Although OsCUC1 and OsCUC3 function together in boundary specification and SAM maintenance, their functions remain different in other developmental processes. Loss of function of *OsCUC1* leads to a dwarf plant structure, pollen defection and twisted‐rolling leaves, while *oscuc3* mutants show no defects in these related developmental processes. Our study indicates that only OsCUC1 – but not OsCUC3 or the other two NAC proteins (OMTN4 and OMTN6) – interacts with the leaf‐rolling related protein CLD1. These results are consistent with the fact that *oscuc1* mutants but not *oscuc3* or *omtn4*/*6* mutants show defects in leaf development. Thus, the different interaction behaviors of OsCUC1 and OsCUC3 with the other gene may be partly responsible for the functional divergence of these two proteins.

### Despite divergence, the mechanisms of meristem/organ boundary specification between Arabidopsis and rice are conserved

**Fig. 8:**
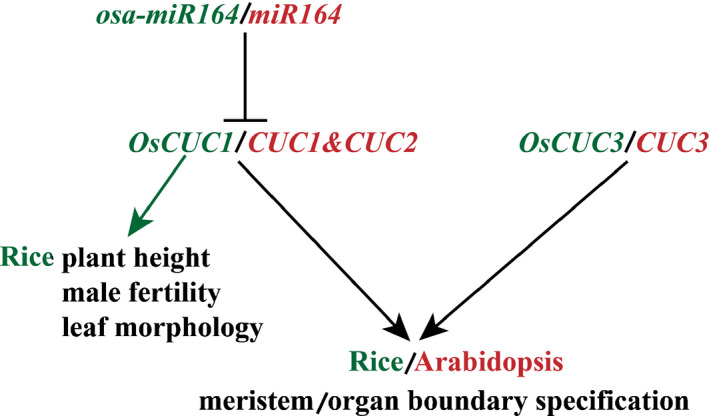
Interplay of a microRNA and *CUP‐SHAPED COTYLEDON* genes controls meristem/organ boundary specification in rice and Arabidopsis. The microRNA (*osa‐miR164*/*miR164*) negatively regulates the *CUP‐SHAPED COTYLEDON* genes (*OsCUC1*/*CUC1*&*CUC2*), which function redundantly with another NAC gene family member (*OsCUC3*/*CUC3*) in meristem/organ boundary specification in both rice and Arabidopsis. In addition, *OsCUC1* controls plant height, male fertility and leaf morphology in rice. Red represents Arabidopsis, and green represents rice.

In Arabidopsis, due to the redundant function of *CUC1* and *CUC2*, neither the *cuc1* nor the *cuc2* single mutation results in a severe phenotype, but the *cuc1 cuc2* double mutation causes a complete lack of embryonic shoot meristem formation. The regenerated *cuc1 cuc2* mutant exhibits abnormal flowers, in which all the sepals and most of the stamens are severely fused (Aida *et al*., 1997, 1999; Takada *et al*., 2001). In rice, the *oscuc1* single mutation causes severe defects in meristem/organ boundary specification, resulting in a reduction in stamen numbers and fusion of leaves/filaments. Consistent with this, systematic sequence analysis revealed that *OsCUC1* is the only homolog of both *CUC1* and *CUC2* (Kondou *et al*., 2009), suggesting that the function of *OsCUC1* may be executed redundantly by *CUC1* and *CUC2* in Arabidopsis. Despite the divergence between *OsCUC1* and *CUC1/2*, the mechanisms of meristem/organ boundary specification are conserved between the dicot Arabidopsis and the monocot *O. sativa* (Fig. 8): one or more microRNA (*osa‐miR164*/*miR164*) target(s) (*OsCUC1*/*CUC1*&*CUC2*) function redundantly with another NAC gene family member (*OsCUC3*/*CUC3*) in meristem/organ boundary specification. In addition, OsCUC1 is involved in leaf development by affecting the stability of CLD1 in the nucleus.

## Author contributions

JJ planned and designed the research. JW, JB, BZ, ML and XL performed experiments. JJ wrote the manuscript. All authors commented on the manuscript. JW and JB contributed equally to this work.

## Supporting information


**Fig. S1** Original blot images from this study.
**Fig. S2** Sequence analysis for OsCUC1 and OsCUC3.
**Fig. S3** Loss‐of‐function of OsCUC1 and OsCUC3 generated by CRISPR/Cas9 in rice.
**Fig. S4** Boundary specification defects in the vegetative growing stage of the rice *oscuc1‐*KO1 mutant.
**Fig. S5** Pollen defects in the rice *oscuc1*‐KO1 mutant.
**Fig. S6** Phenotypes of heterozygous mutants of *OsCUC1* and *OsCUC3* in rice.
**Fig. S7** Dimerization of the Arabidopsis CUC proteins.
**Fig. S8** The development of rice *oscuc1 oscuc3* homozygous double mutants is arrested at the seedling stage.
**Fig. S9** Transcript evidence and expression pattern for rice *osa‐miR64c*.
**Fig. S10** The expression patterns for the other five *osa‐miR164* targets in rice.
**Fig. S11** Knocking out *OMTN4* or *OMTN6* does not lead to defects either in boundary specification or leaf development in rice.
**Fig. S12** The *CLD1* expression level does not significantly change in the rice *oscuc1* mutant.
**Fig. S13** CLD1 does not interact with OsCUC3, OMTN4 or OMTN6 in rice.
**Table S1** The accession numbers of the proteins listed in the phylogenetic tree.
**Table S2** The primer sequences used in this study.
**Table S3** Percentages of aberrant florets of *oscuc1*‐KO and *oscuc3*‐KO plants.
**Table S4** Percentages of aberrant florets of *omtn4‐*KO and *omtn6*‐KO plants.
Please note: Wiley Blackwell are not responsible for the content or functionality of any Supporting Information supplied by the authors. Any queries (other than missing material) should be directed to the *New Phytologist* Central Office.
