# Supplementary material for: The osa‐miR164 target OsCUC1 functions redundantly with OsCUC3 in controlling rice meristem/organ boundary specification
Source: New Phytol. 2020 Oct 25;229(3):1566–81. doi: 10.1111/nph.16939 (PMC7821251; doi:10.1111/nph.16939)
Supplement: Supplementary file 1 — Fig. S1 Original blot images from this study. Fig. S2 Sequence analysis for OsCUC1 and OsCUC3. Fig. S3 Loss‐of‐function of OsCUC1 and OsCUC3 generated by CRISPR/Cas9 in rice. Fig. S4 Boundary specification defects in the vegetative growing stage of the rice oscuc1‐KO1 mutant. Fig. S5 Pollen defects in the rice oscuc1‐KO1 mutant. Fig. S6 Phenotypes of heterozygous mutants of OsCUC1 and OsCUC3 in rice. Fig. S7 Dimerization of the Arabidopsis CUC proteins. Fig. S8 The development of rice oscuc1 oscuc3 homozygous double mutants is arrested at the seedling stage. Fig. S9 Transcript evidence and expression pattern for rice osa‐miR64c. Fig. S10 The expression patterns for the other five osa‐miR164 targets in rice. Fig. S11 Knocking out OMTN4 or OMTN6 does not lead to defects either in boundary specification or leaf development in rice. Fig. S12 The CLD1 expression level does not significantly change in the rice oscuc1 mutant. Fig. S13 CLD1 does not interact with OsCUC3, OMTN4 or OMTN6 in rice. Table S1 The accession numbers of the proteins listed in the phylogenetic tree. Table S2 The primer sequences used in this study. Table S3 Percentages of aberrant florets of oscuc1‐KO and oscuc3‐KO plants. Table S4 Percentages of aberrant florets of omtn4‐KO and omtn6‐KO plants. Please note: Wiley Blackwell are not responsible for the content or functionality of any Supporting Information supplied by the authors. Any queries (other than missing material) should be directed to the New Phytologist Central Office. [file NPH-229-1566-s001.pdf]

New Phytologist

New Phytologist Supporting Information

Article title: The *osa-miR164* target *OsCUC1* functions redundantly with *OsCUC3* in controlling rice meristem/organ boundary specification

Authors: Jun Wang[#], Jinlin Bao[#], Beibei Zhou, Min Li, Xizhi Li, Jian Jin[*]

Article acceptance date: 5 September 2020

The following Supporting Information is available for this article:

Figure S1. The original blot images in this study.

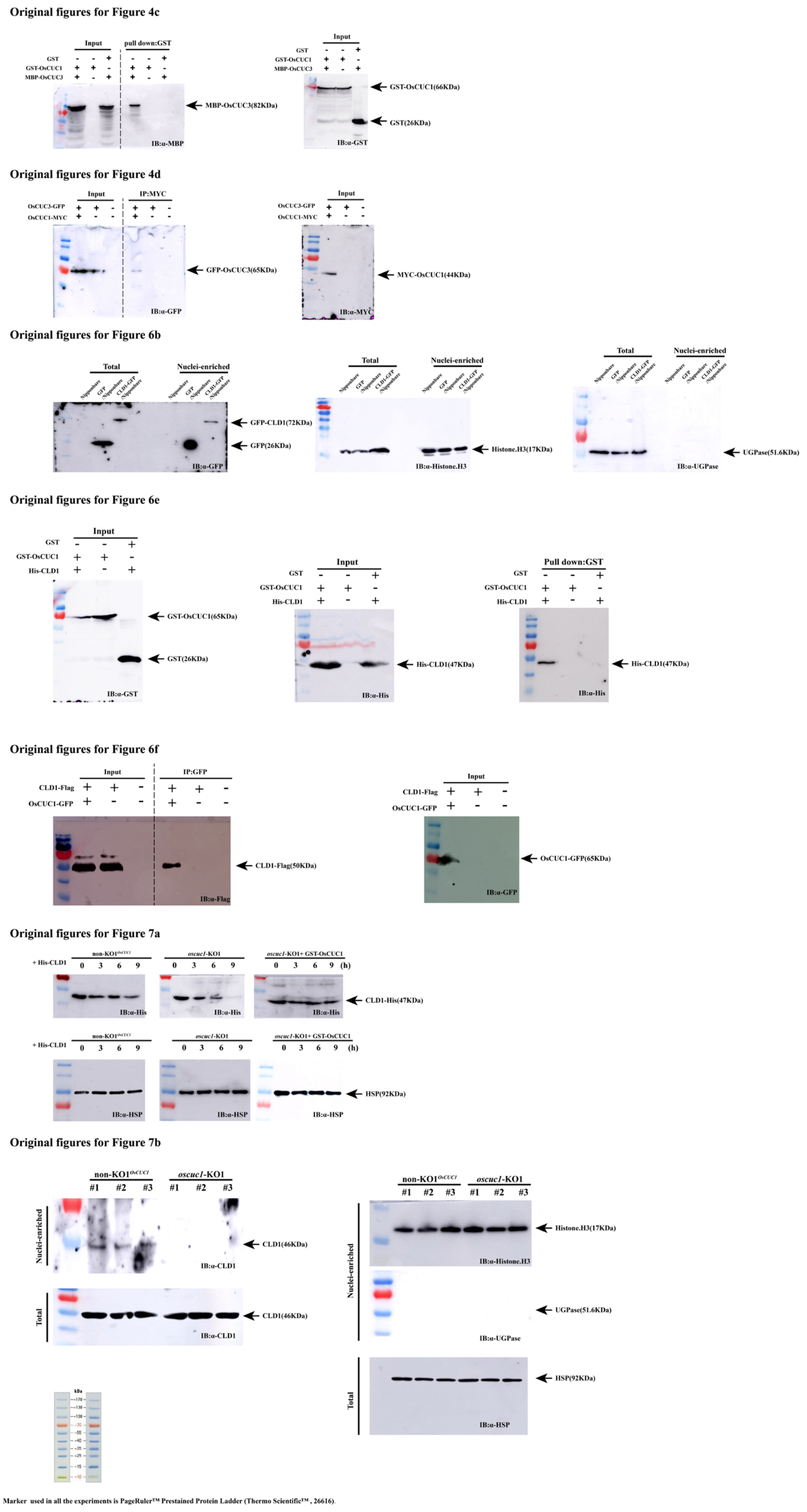

Fig S2. Sequence analysis of OsCUC1 and OsCUC3. (a) Sequence alignment of OsCUC1 and CUC1. (b) Sequence alignment of OsCUC3 and CUC3. The NAC domain is showed in the orange frame, the amino acid corresponding to *miR164* binding region (miR164BR) is showed in green frame. (c) Phylogenetic tree analysis of rice OsCUC1, OsCUC3 and their homologous from *Arabidopsis thaliana*, *Oryza sativa*, *Fragaria vesca*, *Solanum lycopersicum*, *Hordeum vulgare*, *Glycine max*, *Triticum aestivum*, *Rosa chinensis*, *Actinidia deliciosa*, *Citrus clementine*, *Vitis vinifera*, *Petunia hybrid* and *Populus trichocarpa*. Gene IDs are shown. The numbers indicate the bootstrap values calculated from 1000 replicates analyses.

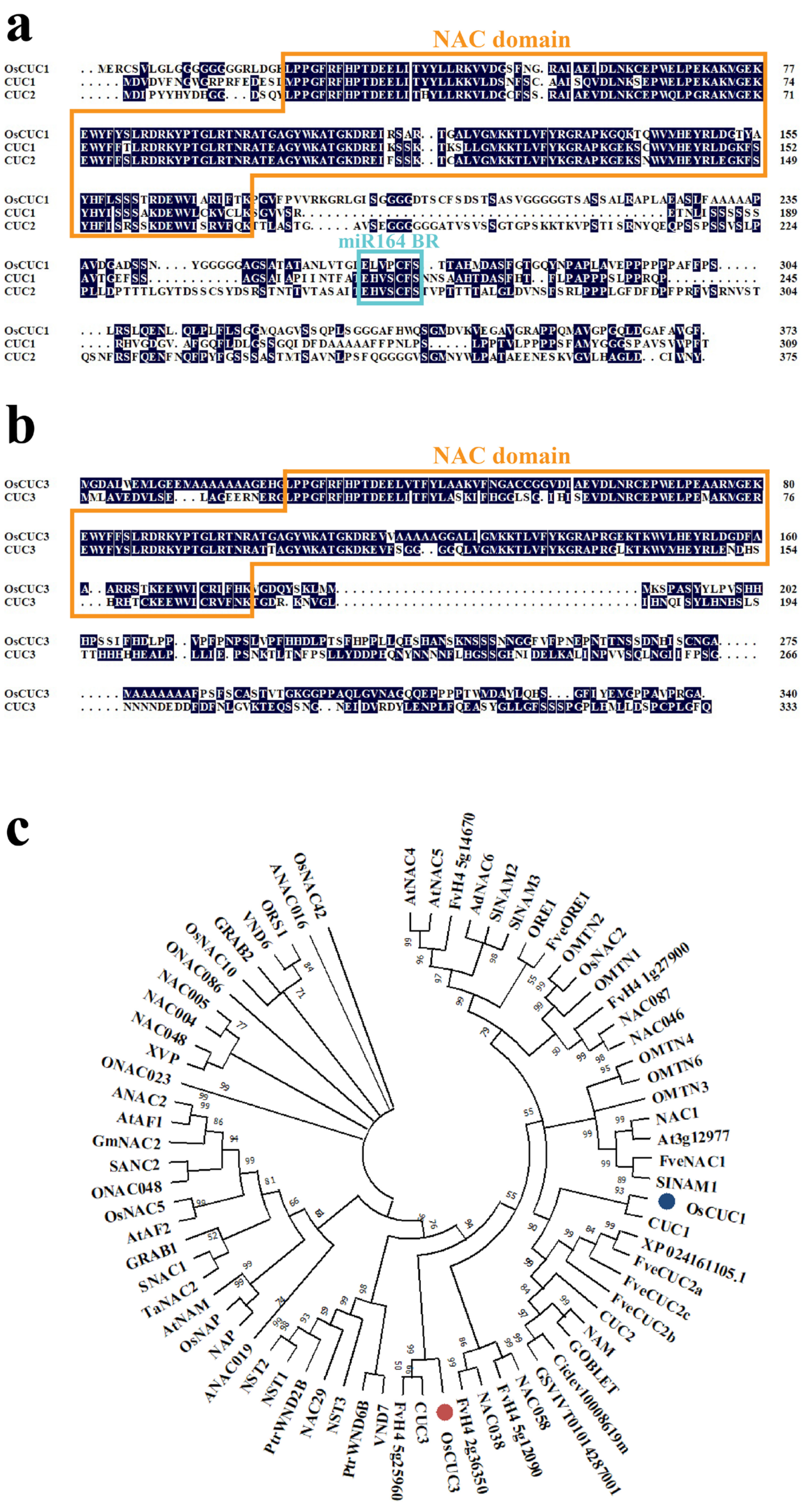
a
NAC domain
miR164 BR
OsCUC1
CUC1
CUC2
b
NAC domain
OsCUC3
CUC3
c
AtNAC4
AtNAC5
FvH4 5g14670
AdNAC6
SlNAM2
SlNAM3
ORE1
FveORE1
OMTN2
OsNAC2
OMTN1
FvH4 1g27900
NAC087
NAC046
OMTN4
OMTN6
OMTN3
NAC1
At3g12977
FveNAC1
SlNAM1
OsCUC1
CUC1
XP 024161105.1
FveCUC2a
FveCUC2c
FveCUC2b
CUC2
NAM
GOBLET
Ciclev10008619m
GSVIVT01014287001
NAC058
FvH4 5g12090
NAC038
FvH4 2g36350
OsCUC3
CUC3
FvH4 5g25960
VND7
PtrWND6B
NST3
NAC29
PtrWND2B
NST1
NST2
ANAC019
NAP
OsNAP
AtNAM
TaNAC2
SNAC1
GRAB1
AtAF2
OsNAC5
ONAC048
SANC2
GmNAC2
AtAF1
ANAC2
ONAC023
XVP
NAC048
NAC004
NAC005
ONAC086
OsNAC10
GRAB2
VND6
ORS1
ANAC016
OsNAC42

Fig S3. Loss-of-function of OsCUC1 and OsCUC3 generated by CRISPR/Cas9 in rice. (a) The *oscuc1*-KO1 harbors 1bp deletion leading to a frame-shift with premature transcription termination, the *oscuc1*-KO2 harbors 1bp insertion leading to a frame-shift with premature transcription termination. (b) Both *oscuc3*-KO1 and *oscuc3*-KO2 harbor 1 bp insertion at different sites of the coding region, result in a premature stop codon. The letters with colorful background indicate the identical amino acids with the wild type proteins.

a

```
OsCUC1      MERCSVLGLGGGGGGGGRLDGELPPGFRFHPTDEELITYYLLRKVVDGSFNGRAIAEIDL   60
oscuc1-KO1  MERCSVLGLGGGGGGGGRLDGELPPGFRFHPTDEELITYYLLRKVVDGSFNGRAIAEIDL   60
oscuc1-KO2  MERCSVSGAGRWRGRGRAAGRRAAAGVPVPPDGRGADHLLPAAEGGGRELQRARHRGDRP   60

OsCUC1      NKCEPWELPEKAKMGEKEWYFYSLRDRKYPTGLRTNRATGAGYWKATGKDREIRSARTGA  120
oscuc1-KO1  NKCEPWELPEKAKMGEKEWYFYSLRDRKYPT....DSAPTAPRAPATGRP.PARTARS..  113
oscuc1-KO2  EQVRAVGAAGEGQDGGEGVVLLQPPRPQVPHG..TPHQPRHGRRLLEGHRQGPRDPQR..  116

OsCUC1      LVGMKKTLVFYRGRAPKGQKTQWVMHEYRLDGTYAYHFLSSSTRDEWVIARIFTKPGVFP  180
oscuc1-KO1  ............................................................  113
oscuc1-KO2  ............................................................  116

OsCUC1      VVRKGRLGISGGGGDTSCFSDSTSASVGGGGGTSASSALRAPLAEASLFAAAAAPAVDGA  240
oscuc1-KO1  ..................................................AAPAPAPSSA  123
oscuc1-KO2  ..................................................PHRRPRRHEE  126

OsCUC1      DSSNYGGGGGAGSATATANLVTGLELVPCFSTTAHMDASFGTGQYNPAPLAVEPPPPPPA  300
oscuc1-KO1  ............................................................  123
oscuc1-KO2  DPRLLPRPRPQGPEDPVG........................................HA  146

OsCUC1      FFPSLRSLQENLQLPLFLSGGMQAGVSSQPLSGGGAFHWQSGMDVKVEGAVGRAPPQMAV  360
oscuc1-KO1  ............................................................  123
oscuc1-KO2  RVPPRRHLRLPLPLLLHPGG........................................  166

OsCUC1      GPGQLDGAFAWGF                                                 373
oscuc1-KO1  .............                                                 123
oscuc1-KO2  .............                                                 166
```

b

```
OsCUC3      MGDALWEMLGEEMAAAAAAAGEHGLPPGFRFHPTDEELVTFYLAAKVFNGACCGGVDIAE   60
oscuc3-KO1  MGDALWEMLGEEMAAAAAAAGEHGLPPGFRFHPTDEELVTFYLAAKVFNGACCGGVDIAE   60
oscuc3-KO2  MGDALWEMLGEEMAAAAAAAGETRAAAGVQVPPHRRGARHLLPRRQGVQRRVLRRRGHRG   60

OsCUC3      VDLNRCEPWELPEAARMGEKEWYFFSLRDRKYPTGLRTNRATGAGYWKATGKDREVVAAA  120
oscuc3-KO1  VDL..................................................KPVRAVG   70
oscuc3-KO2  GGP..................................................EPVRAVG   70

OsCUC3      AAGGALIGMKKTLVFYKGRAPRGEKTKWVLHEYRLDGDFAAARRSTKEEWVICRIFHKVG  180
oscuc3-KO1  AAGGGEDGGEGVVLLQP...PRPQVPDGAAHQPRHRRRLLEGHRQGQGGGRRRRRRRRAH  127
oscuc3-KO2  AAGGGEDGGEGVVLLQP...PRPQVPDGAAHQPRHRRRLLEGHRQGQGGGRRRRRRRRAH  127

OsCUC3      DQYSKLMMMKSPASYYLPVSHHHPSSIFHDLPPVPFPNPSLVPFHHDLPTSFHPPLLQHS  240
oscuc3-KO1  RHEEDARLLQGPR....PARRED...........................QVGPPRVPPR  156
oscuc3-KO2  RHEEDARLLQGPR....PARRED...........................QVGPPRVPPR  156

OsCUC3      HANSKNSSSNNGGFVFPNEPNTTNSSDNHISCNGAMAAAAAAAFPSFSCASTVTGKGGPP  300
oscuc3-KO1  RRLRRRSPLHQGGMGDLQDLSQGRRSVQQADDD........................EEP  192
oscuc3-KO2  RRLRRRSPLHQGGMGDLQDLSQGRRSVQQADDD........................EEP  192

OsCUC3      AQLGVNAGQQEPPPPTWMDAYLQHSGFIYEMGPPAVPRGA                      340
oscuc3-KO1  SQLLP...PSEPPPPQQHLP....................                      209
oscuc3-KO2  SQLLP...PSEPPPPQQHLPP...................                      210
```

Fig S4. Boundary specification defects in vegetative growing stage of the rice *oscuc1*-KO1 mutant. (a) and (b) Histologic slides (cross section) of leaf blade (the basal of the leaf blade) for *oscuc1*-KO1 and its non-KO control. (c) and (d) Histologic slides (cross section) of leaf sheath (1 cm below the flag leaf) for *oscuc1*-KO1 and its non-KO control. Scale bars = 500 μm.

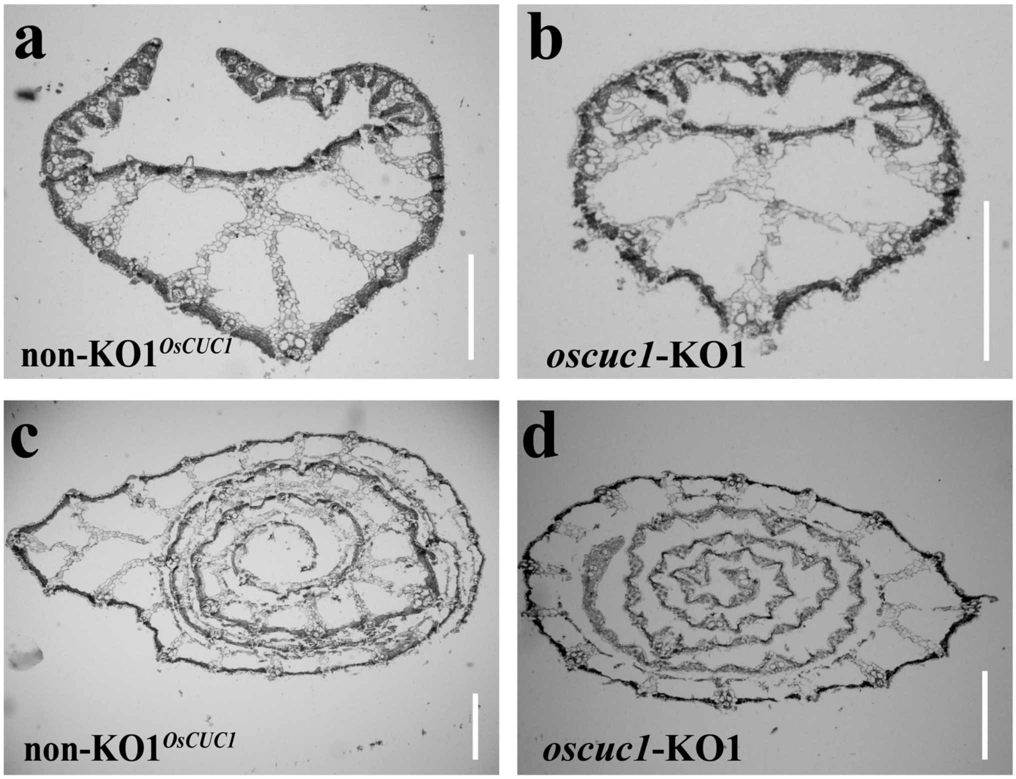

Fig S5. Pollen defects of the rice *oscuc1*-KO1 mutant. (a) and (b) Pollen viability test for non-KO^{OsCUC1} and *oscuc1*-KO1 plant, represents the pollen activity test for 3 biological replicates of each line respectively. Scale bar = 500 μm.

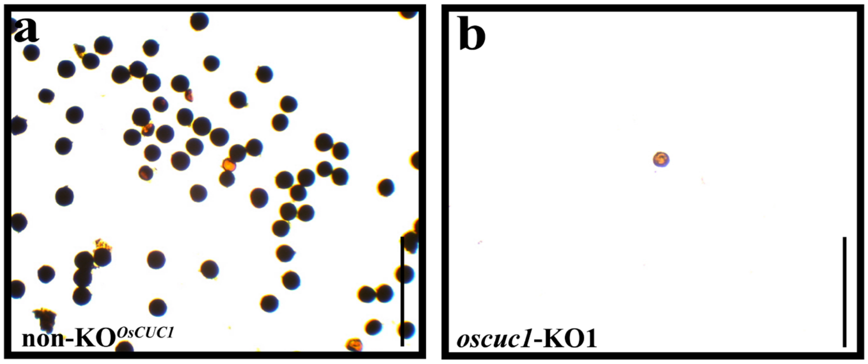

Fig S6. Phenotypes of heterozygous mutants of *OsCUC1* and *OsCUC3* in rice. (a) and (b) The panicle of T_0 non-KO^{OsCUC1} and *oscuc1*-KO1. (c) Self-pollinated florets of T_0 *oscuc1*-KO1. (d) Cross-pollinated florets which pollen from Nipponbare was used to pollinate the gynoecia of *oscuc1*-KO1. (e) Detail of the pane in (d). (f) and (g) F_2 generation of non-KO1^{OsCUC1}, *oscuc1*-KO1/+ and *oscuc1*-KO1 in seedling stage and heading stage, all of these plants were derived from a F_1 *oscuc1*-KO1/+ plant. (h) and (i) non-KO1 ^{OsCUC3}, *oscuc3*-KO1/+ and *oscuc3*-KO1 in seedling stage and heading stage, all of these plants were derived from a *oscuc3*-KO1/+ plant.

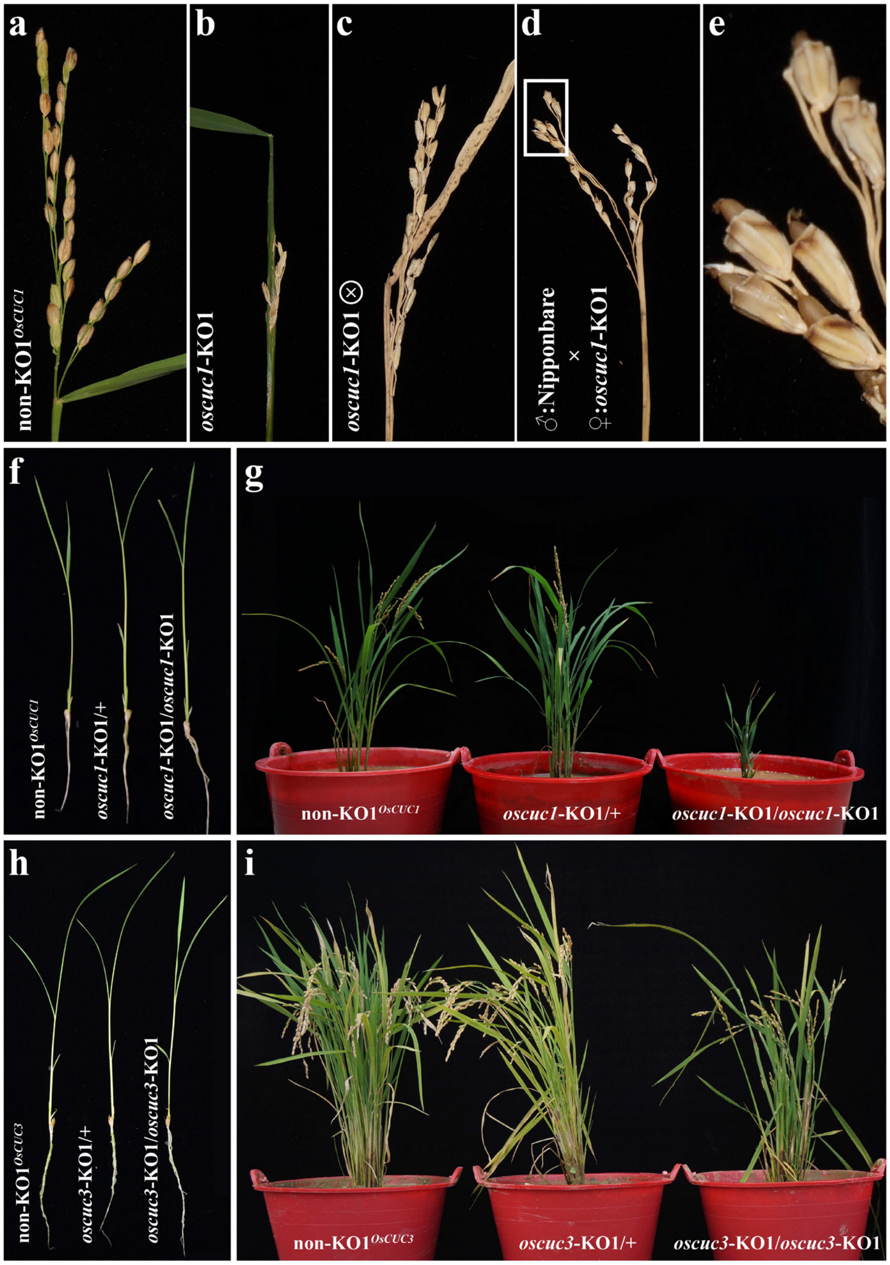

Fig S7. Dimerization of the Arabidopsis CUC proteins. (a) BiFC assay indicated that the CUC1, CUC2 and CUC3 do not form heterodimers or homodimers. Interaction between AP1 and SEP3 was used as positive control. (b) The schematic of constructing *mCUC1* and *mCUC2*. The red letters indicate mutant nucleotides introduced into the *miR164* target site in the *mCUC1* or *mCUC2* construction, which interrupts the *miR164* target site without changing the amino acid residues. Watson-Crick base pairing between the mRNA and *miR164* is indicated by black lines. Mismatches and G:U wobbles are indicated by a star or equals sign, respectively. (c) BiFC assay indicated the homodimerization of mCUC1 and mCUC2, and the heterodimerization of mCUC1-mCUC2, mCUC1-CUC3 and mCUC2-CUC3. Co-expression of GFP_C-mCUC1 plus GFP_N or GFP_C plus GFP_N-mCUC1 or GFP_C-mCUC2 plus GFP_N or GFP_C plus GFP_N-mCUC2 or GFP_C-CUC3 plus GFP_N or GFP_C plus GFP_N-CUC3 were used as negative controls. Scale bars = 5 µm.

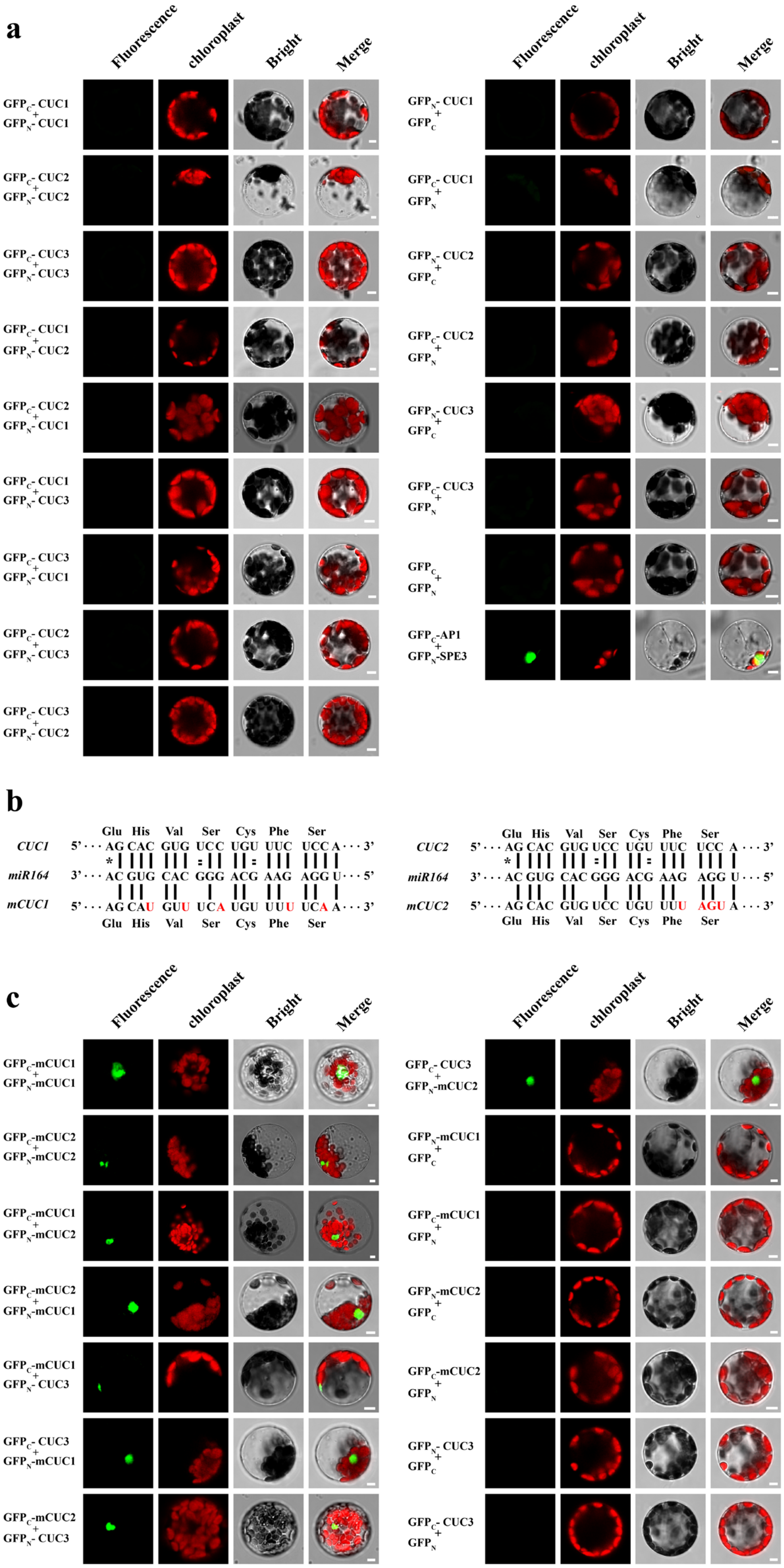
a
Fluorescence
chloroplast
Bright
Merge
GFPC- CUC1 + GFPN- CUC1
GFPC- CUC2 + GFPN- CUC2
GFPC- CUC3 + GFPN- CUC3
GFPC- CUC1 + GFPN- CUC2
GFPC- CUC2 + GFPN- CUC1
GFPC- CUC1 + GFPN- CUC3
GFPC- CUC3 + GFPN- CUC1
GFPC- CUC2 + GFPN- CUC3
GFPC- CUC3 + GFPN- CUC2
GFPN- CUC1 + GFPC
GFPC- CUC1 + GFPN
GFPN- CUC2 + GFPC
GFPC- CUC2 + GFPN
GFPN- CUC3 + GFPC
GFPC- CUC3 + GFPN
GFPC + GFPN
GFPC-AP1 + GFPN-SPE3
b
Glu His Val Ser Cys Phe Ser
CUC1 5'··· AG CAC GUG UCC UGU UUC UCC A···3'
miR164 3'··· AC GUG CAC GGG ACG AAG AGG U···5'
mCUC1 5'··· AG CAU GUU UCA UGU UUU UCA A···3'
Glu His Val Ser Cys Phe Ser
CUC2 5'··· AG CAC GUG UCC UGU UUC UCC A···3'
miR164 3'··· AC GUG CAC GGG ACG AAG AGG U···5'
mCUC2 5'··· AG CAC GUG UCC UGU UUU AGU A···3'
c
GFPC-mCUC1 + GFPN-mCUC1
GFPC-mCUC2 + GFPN-mCUC2
GFPC-mCUC1 + GFPN-mCUC2
GFPC-mCUC2 + GFPN-mCUC1
GFPC-mCUC1 + GFPN- CUC3
GFPC- CUC3 + GFPN-mCUC1
GFPC-mCUC2 + GFPN- CUC3
GFPC- CUC3 + GFPN-mCUC2
GFPN-mCUC1 + GFPC
GFPC-mCUC1 + GFPN
GFPN-mCUC2 + GFPC
GFPC-mCUC2 + GFPN
GFPN- CUC3 + GFPC
GFPC- CUC3 + GFPN

Fig S8. The development of rice *oscuc1 oscuc3* homozygous double mutants is arrested in the seedling stage. (a) to (d) Histologic slides (vertical section) of SAM for wild type (Nipponbare), *oscuc1*, *oscuc3* and *oscuc1 oscuc3*. Scale bars = 50 µm. (e) to (h) Appearance of *oscuc1/+ oscuc3/ oscuc3* and three *oscuc1 oscuc3* homozygous double mutants in 15 DAG (day after germination). (i) to (l) Appearance of the corresponding plants in 36 DAG (day after germination). Scale bars = 1 cm.

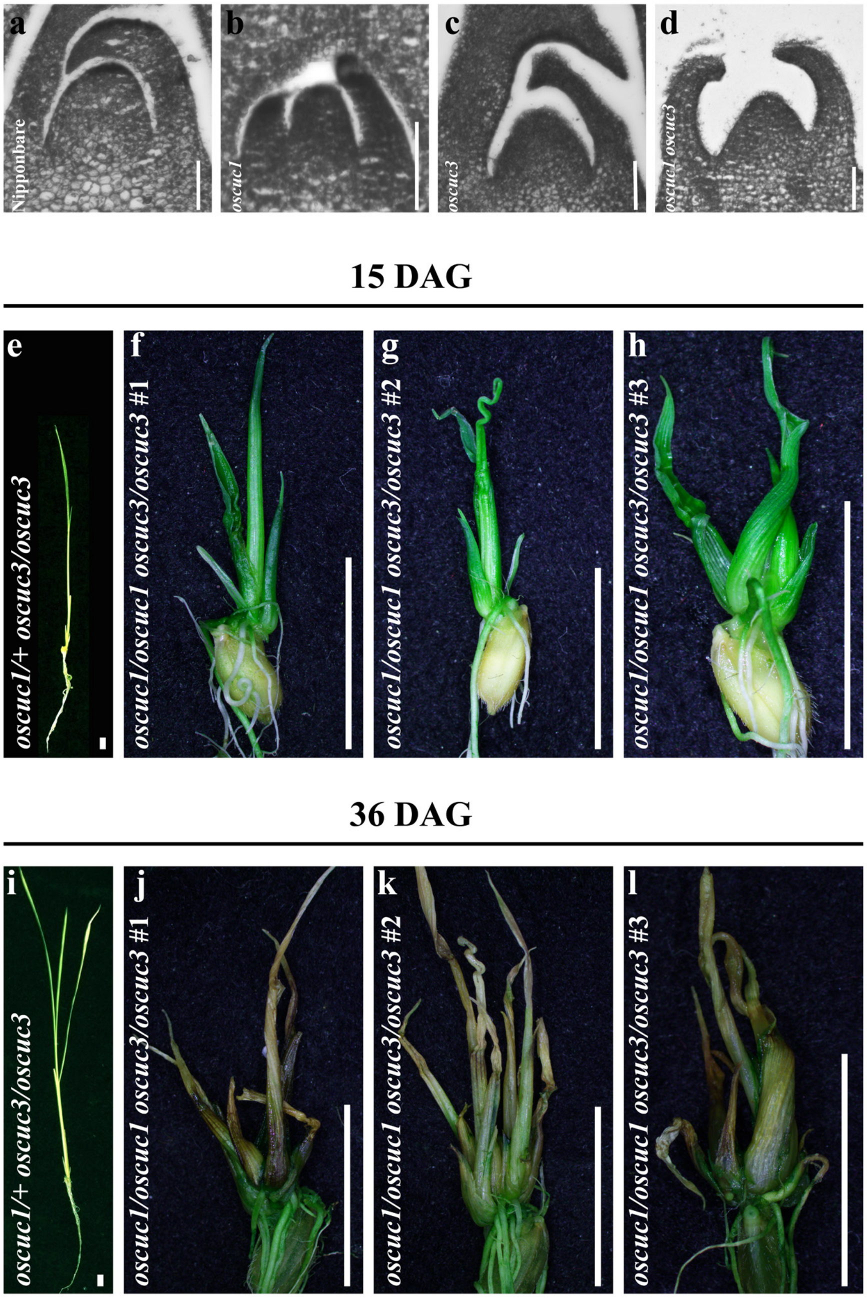

Fig S9. Transcript evidence and expression pattern of rice *osa-miR64c*. (a) RT-PCR with specific primers for *pre-osa-miR164c* using the Nipponbare cDNA as the templates. The product from reverse transcription without reverse transcriptase was used as negative control in the RT-PCR. Three biological replicates were used in this assay. (b) The relative expression level of *pre-osa-miR164c* in seedling (3 days), leaf, root, UBI (unelongated basal internode), leaf sheath, bract, flag leaf, YP 0.5 cm (young panicle 0.5 cm), YP 2 cm (young panicle 2 cm), YP 8 cm (young panicle 8 cm), stamen, pistil, palea and lemma. Values are shown as means ± s.d. (n = 3).

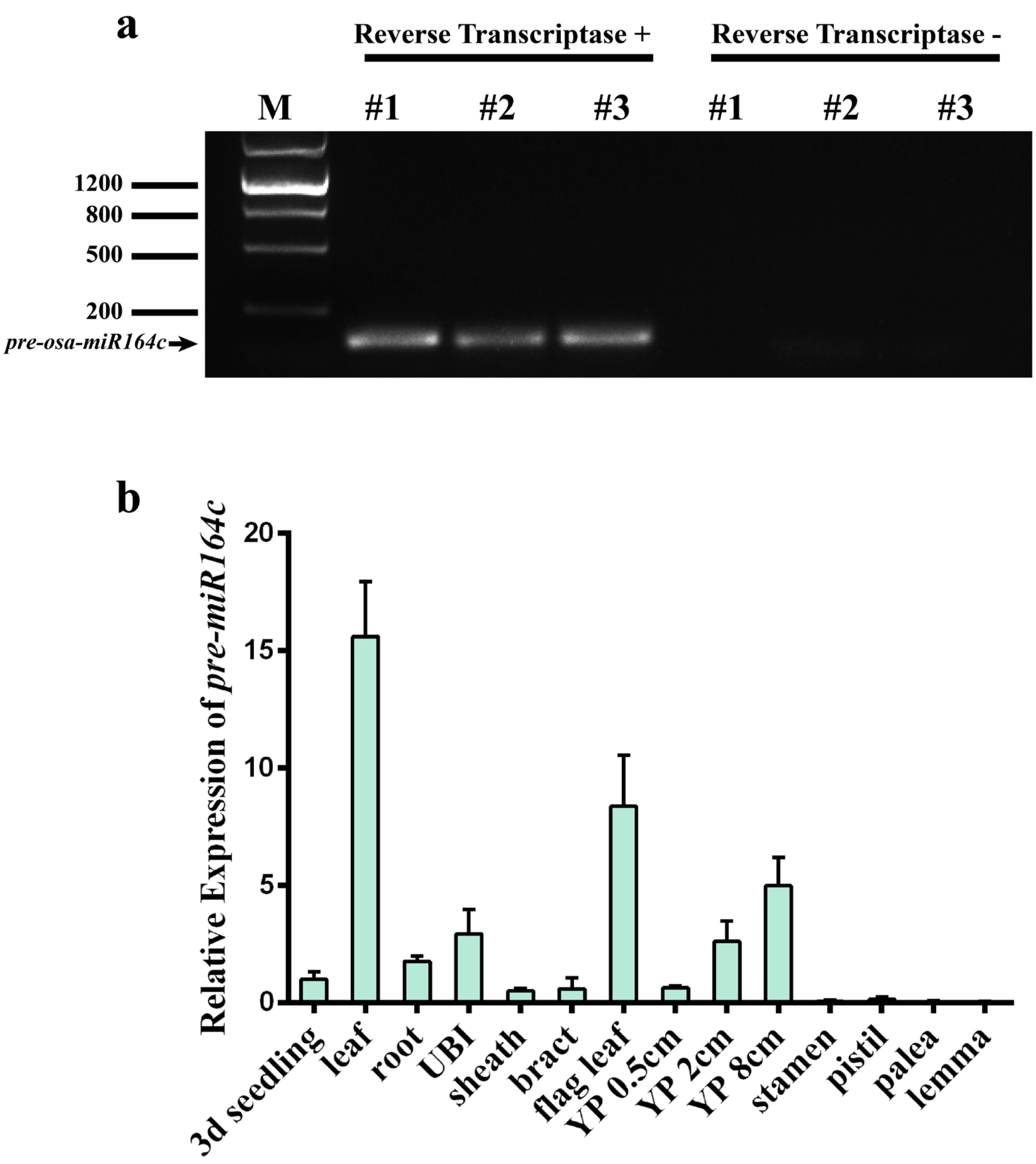

Fig S10. The expression patterns of the other five *osa-miR164* targets in rice. (a) to (e) The relative expression level of *OMTN1*, *OMTN2*, *OMTN3*, *OMTN4* and *OMTN6* in leaf, root, flag leaf, pistil, stamen, palea and lemma for two individual *OE-osa-miR164c* lines and the empty-vector control line (EVC). Values are shown as means ± s.d. (n = 3).

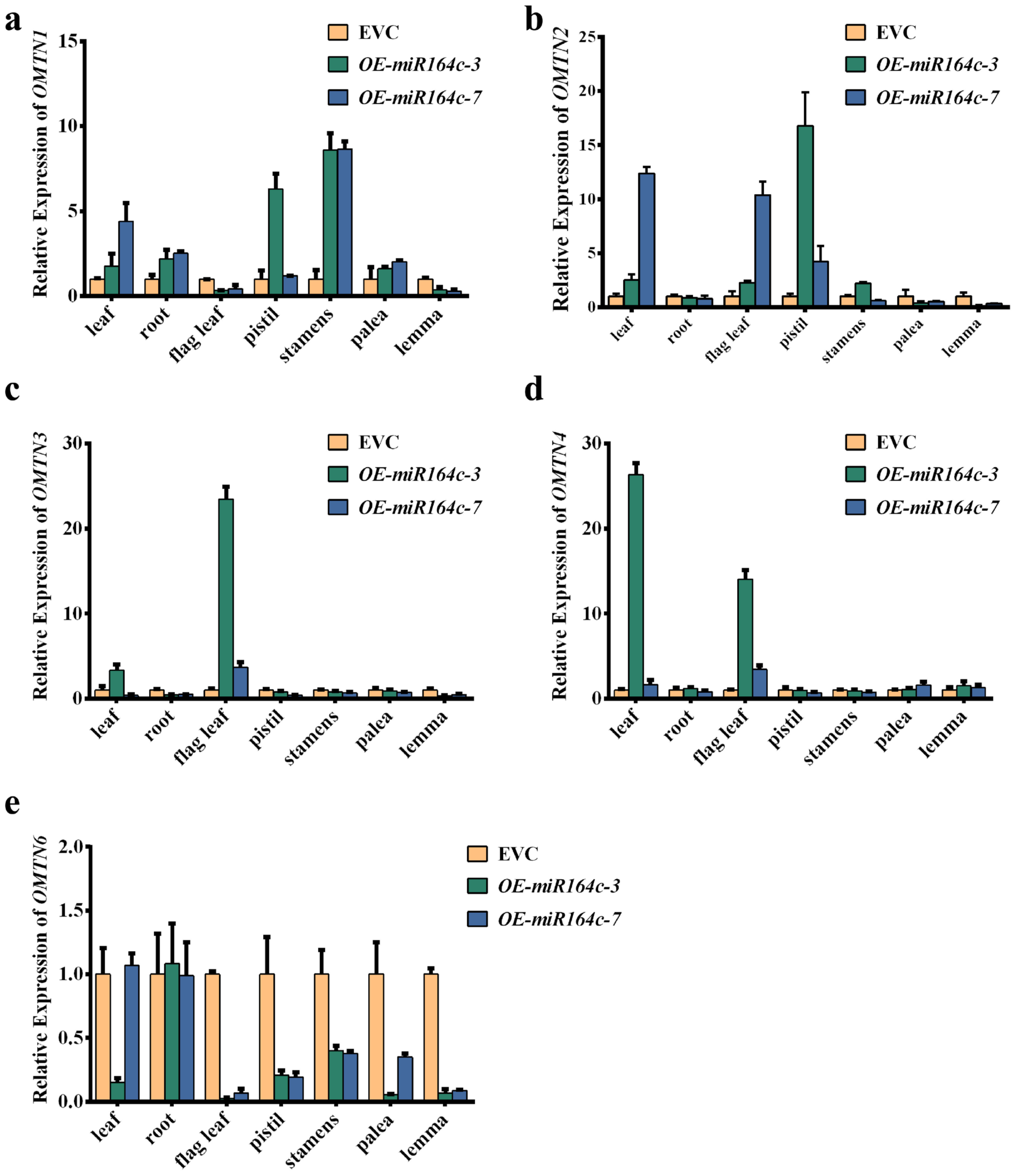

Fig S11. Knocking out *OMTN4* or *OMTN6* dose not lead to defects either in boundary specification or in leaf development in rice. (a) Schematic diagram of *OMTN4* gene structure and the CRISPR/Cas9 target site. The PAM site is underline. The mutant site is shown in red. (b) The phenotype of the *omtn4*-KO1 line and non-KO^{OMTN4} control line. The genetic background of both lines is Nipponbare. (c) The phenotype of the *omtn4*^{ZH11} and control line Zhonghua11 (ZH11). (d) Schematic diagram of *OMTN6* gene structure and the CRISPR/Cas9 target site. The PAM site is underline. The mutant site is shown in red. (e) The phenotype of the *omtn6*-KO1 line and non-KO^{OMTN6} control line. The genetic background of both lines is Nipponbare. (f) The phenotype of the *omtn6*^{ZH11} and control line Zhonghua11.

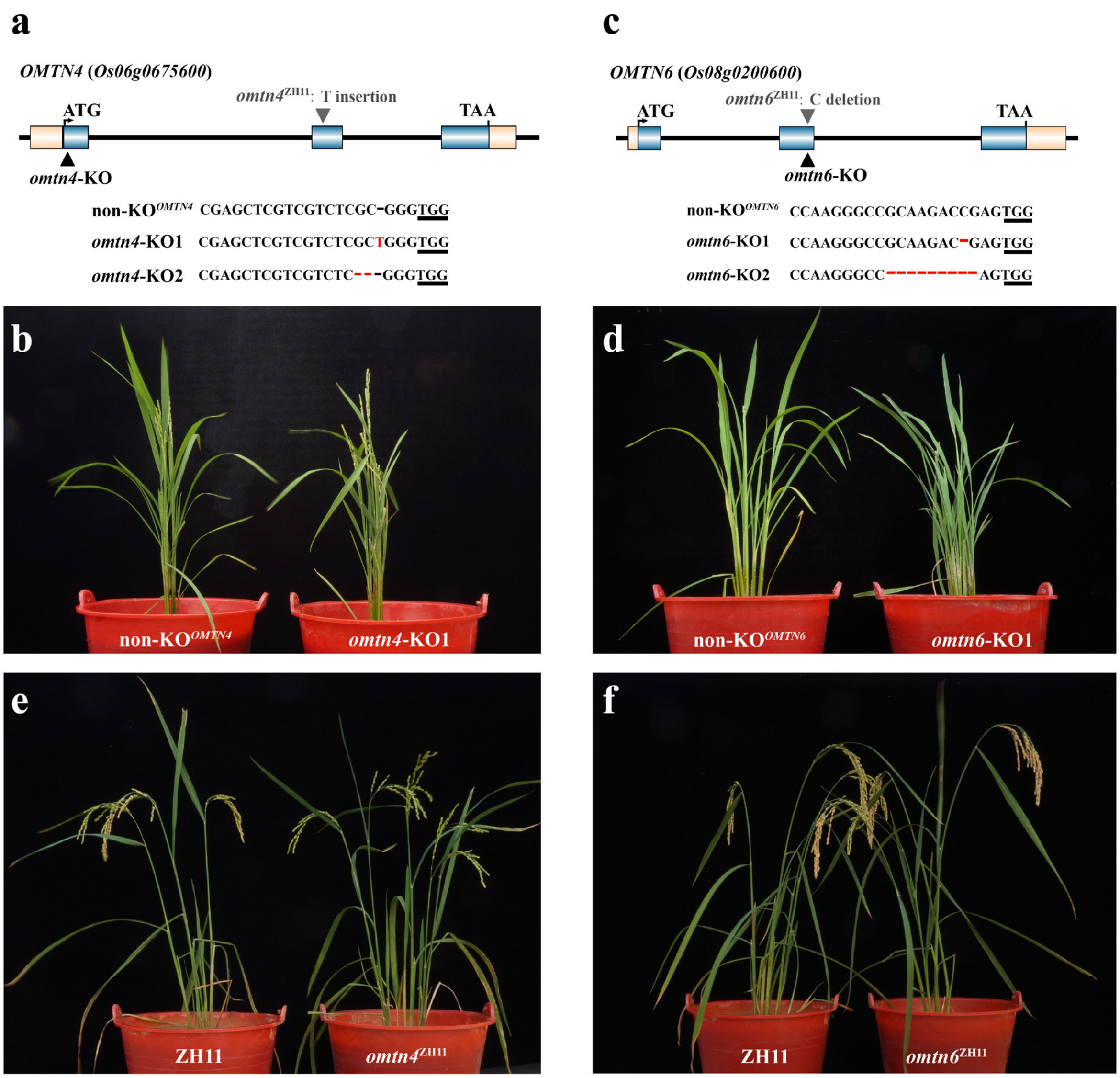

Fig S12. The *CLD1* expression level does not significantly change in the rice *oscuc1* mutant. (a) Structure of *CLD1* gene. The three pairs of arrows indicated the three pairs of primers using for qRT-PCR. (b) The relative expression level of *CLD1* evaluated by three pairs of primers. Values are shown as means ± s.d. (n = 3).

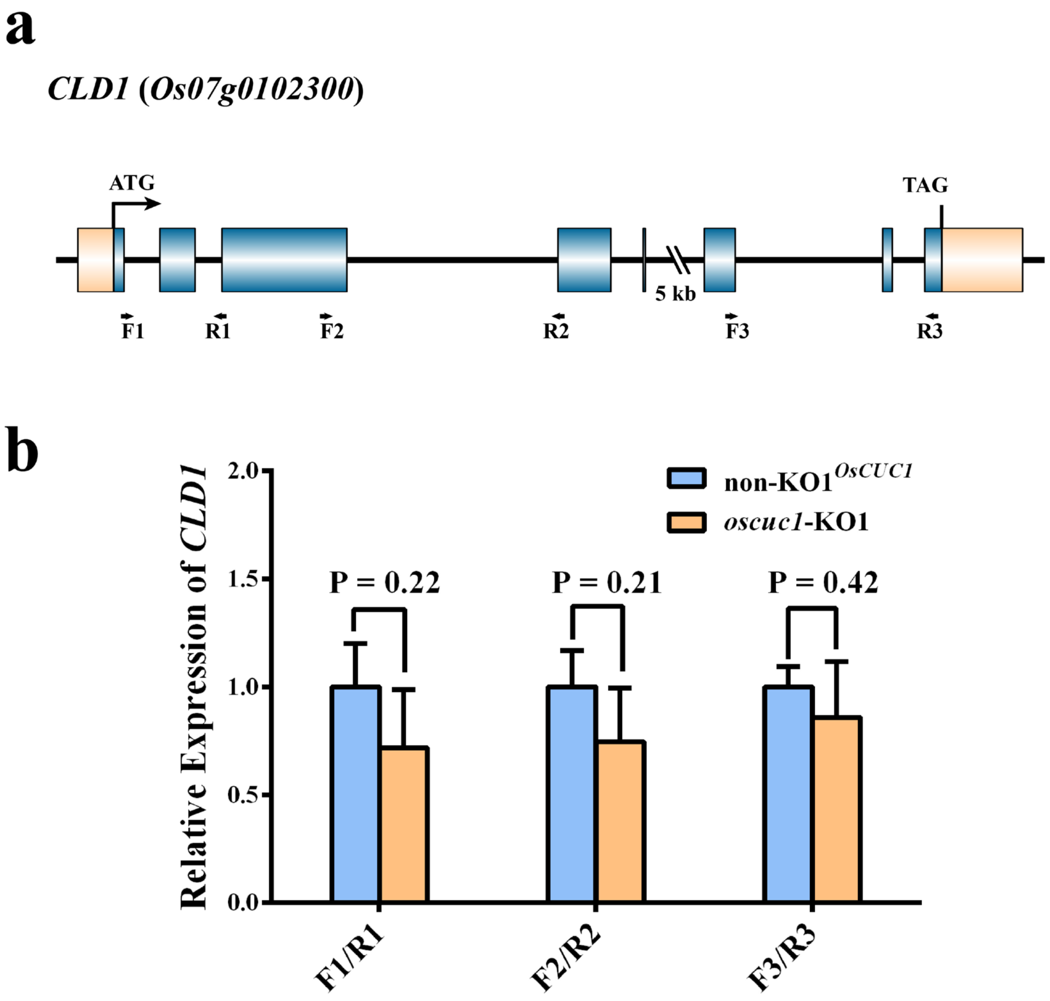

Fig S13. CLD1 does not interact with OsCUC3, OMTN4 or OMTN6 in rice. (a) BiFC assay indicated that CLD1 does not interact with OsCUC3, OMTN4 or OMTN6. Co-expression of GFP_C-OsCUC1 plus GFP_N-CLD1 was used as positive control. Scale bars = 5 μm. (b) Y2H assay indicated that CLD1 does not interact with OsCUC3, OMTN4 or OMTN6. The combination of BK-53 plus AD-T was used as a positive control, while BK-LAM plus AD-T or BK plus AD were used as negative controls.

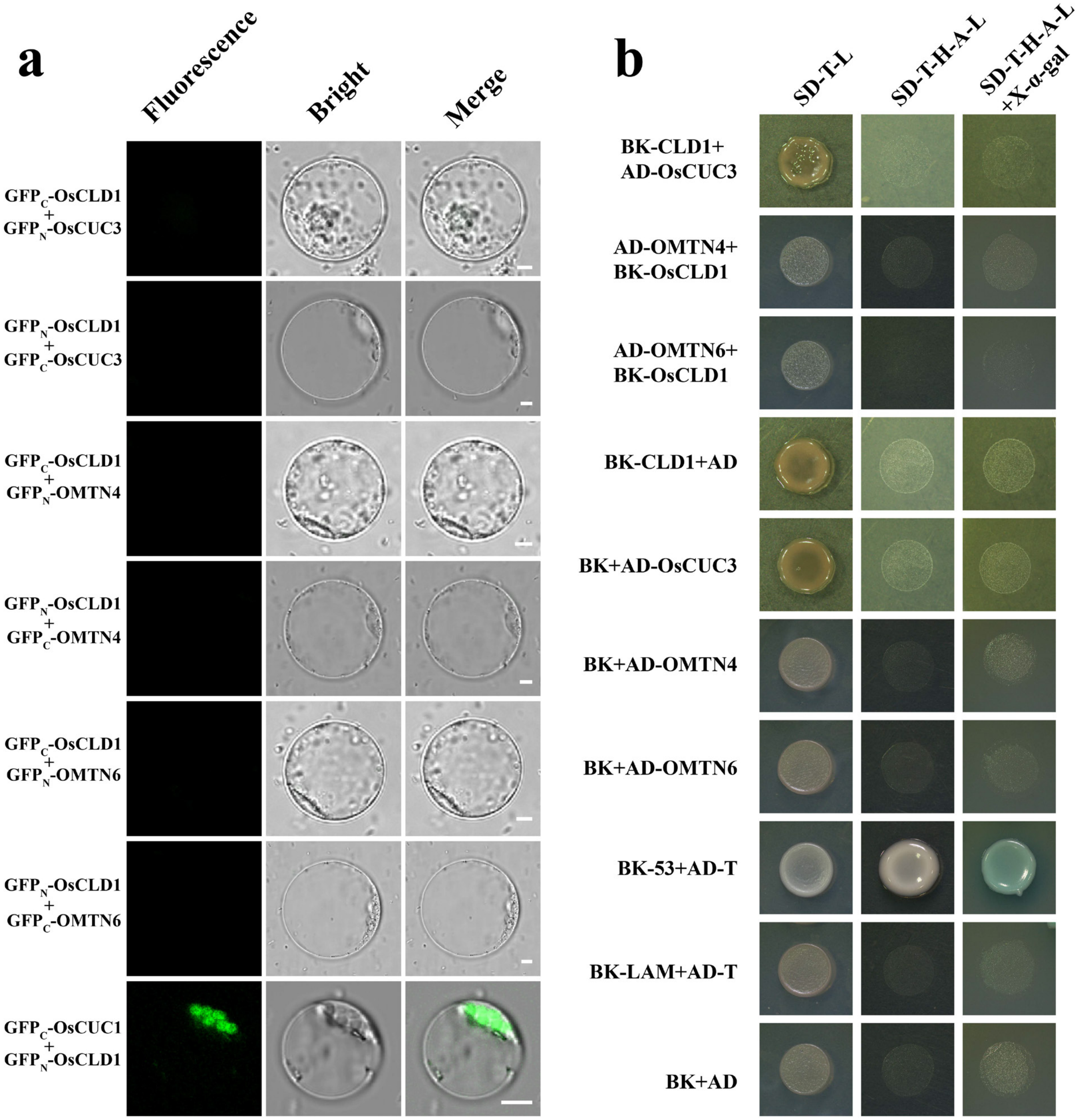

Table S1. The accession numbers for the proteins listed in phylogenetic tree

Species	Protein name	Accession numbers
	CUC1	NP_188135
	CUC2	NP_200206
	CUC3	XP_015648828
	NAC004	NP_171726
	NAC005	NP_171727
	NAC048	AAN38683
	VND6	ABE02409
	VND7	OAP19653
	NAP	NP_564966
	XVP	NP_171725
	ANAC2	NP_171677
	ORE1	NP_198777
	NAC1	AAO64808
	AtNAC4	AAP21227
Arabidopsis thaliana	NAC058	NP_188469
	NAC038	NP_850054
	NAC087	NP_974800
	NAC046	NP_187056
	AtNAC5	NP_001331908
	ORS1	OAP09180
	At3g12977	NP_001327438
	ANAC016	ABO38757
	ANAC019	NP_175697
	NST1	NP_182200
	NST2	OAP06131
	NST3	NP_174554
	AtNAM	NP_175696
	AtAF1	NP_171677
	AtAF2	NP_680161

Oryza sativa	OsCUC1	XP_015642575
	OsCUC3	BAC57407
	OMTN1	XP_015623389
	OMTN2	XP_015633922
	OMTN3	XP_015620576
	OMTN4	XP_025881778
	OMTN6	XP_015648318
	ONAC086	XP_015638590
	NAC29	AEO53047.1
	OsNAP	BAH01634
	OsNAC2	BAG88085.1
	ONAC023	BAG98022.1
	ONAC048	BAG90892.1
	OsNAC5	AB028184.1
	SNAC1	BAG90542.1
	OsNAC10	BAG91345.1
	OsNAC42	BAG90930.1
	SNAC2	BAG90892.1
Fragaria vesca	FveCUC2a	XP_004291499
	FveCUC2b	FvH4_6g06070
	FveCUC2c	FvH4_3g03540
	FvH4_5g25960	XP_004301620
	FvH4_5g12090	XP_004300523
	FvH4_2g36350	XP_004291275
	FvH4_1g27900	XP_004309786
	FvH4_5g14670	XP_004299340
Fragaria vesca	FveORE1	XP_004304286
	FveNAC1	XP_004294208
Solanum lycopersicum	GOBLET	ACL14371
	SlNAM2	solyc03g115850
	SlNAM3	solyc06g069710

Solanum lycopersicum	SlNAM1	solyc07g066330
Hordeum vulgare	GRAB1	KAE8820591
	GRAB2	KAE8816523
Glycine max	GmNAC2	AAY46122
Triticum aestivum	TaNAC2	AAU08786
Rosa chinensis	XP_024161105.1	XP_024161105
Actinidia deliciosa	AdNAC6	AZL19352
Citrus clementina	Ciclev10008619m	ESR65017
Vitis vinifera	GSVIVT01014287001	CBI20259
Petunia hybrida	NAM	X92205
Populus trichocarpa	PtrWND2B	XP_024450691
	PtrWND6B	ADR00341

Table S2. The primer sequences using in this study.

Primer sequences for generating DNA constructs

Experiment	Construct	Primer name	Sequence (5'→3')
overexpressed *osa-miR164c*	*pUbi::osa-miR164c*	OE-miR164cF	AACTGCAGTTGATGCTACTGTAGCCATC
		OE-miR164cR	GGACTAGTTCCTATCATTTGATTCATTG
GUS assay	*pOsCUC1::GUS*	OsCUC1pgus-F	CCCAAGCTTTGCTCTCGACCTGTAGATTCC
		OsCUC1pgus-R	GCTCTAGACCAGCTGCTGCAACAAGTAAC
	pOsCUC1::OsCUC1-GUS	pro-cds-gus-F	ACGCGTCGACTGCTCTCGACCTGTAGATTCC
		pro-cds-gus-R	GCTCTAGAGAAGCCCCATGCAAAGGCGCCG
	pOsCUC1-mOsCUC1-GUS	mOsCUC1-R	CGAAAAACATGGGACCAGTTCAAGGCCGGTGACCAAGTTGGC
		mOsCUC1-F	ACTGGTCCCATGTTTTTCGACCACAGCCCACATGGATGCCTC
Yeast two-hybrid assay	*pGADT7-OsCUC1*	OsCUC1-AD-F	CGGAATTCATGGAGCGGTGCAGCGTGC
		OsCUC1-AD-R	ACGCGTCGACCTAGAAGCCCCATGCAAAG
	pGADT7-OsCUC3	OsCUC3-AD-F	CGGAATTCATGCATCATCACTCGGCCAC
		OsCUC3-AD-R	CGGGATCCTCATGCGCCCCTGGGCACTG
	pGADT7-OMTN4	OMTN4-F	GGAATTCCATATGATGAGCGGGATGAATTCGCTGAGC
		OMTN4-R	CCCTCGAGTCAACTGAGTGAGTTCCACATTTGTGTG
	pGADT7-OMTN6	OMTN6-F	CGGAATTCATGAGTTTCATAGGCATGGTGG
		OMTN6-R	CGGGATCCTTATTGGGGGTTCCACATCTGTGAC
	pGBKT7-CLD1	CLD1-BD-F	GGAATTCCATATGATGGGGATGGCCCCCGCCGGTCG
		CLD1-BD-R	CGGAATTCCTAGACTTGTAGCCAAATAGCAACAAC
Transactivation assay	*pGBKT7-OsCUC1*	OsCUC1-BD-F	CGGAATTCATGGAGCGGTGCAGCGTGC
		OsCUC1-BD-R	CGGGATCCCTAGAAGCCCCATGCAAAG
	pGBKT7-OsCUC3	OsCUC3-BD-F	CGGAATTCATGCATCATCACTCGGCCAC
		OsCUC3-BD-R	CGGGATCCTCATGCGCCCCTGGGCACTG
BiFC assay	*p35S::CLD1-Vc/Vn*	CLD1-Vc/Vn-F	CGGAATTCATGGGGATGGCCCCCGCCGGTCG
		CLD1-Vc/Vn -R	GGAATTCCATATGGACTTGTAGCCAAATAGCAAC
	p35S::OsCUC1-Vc/Vn	OsCUC1-Vc/Vn-F	CGGAATTCATGGAGCGGTGCAGCGTGCTGGGG
		OsCUC1-Vc/Vn -R	GGAATTCCATATGGAAGCCCCATGCAAAGGCGCCG
	p35S::OsCUC3-Vc/Vn	OsCUC3-Vc/Vn-F	CGGGATCCATGCATCATCACTCGGCCACCATGGG
		OsCUC1-Vc/Vn-R	CGGAATTCACTGCGCCCCTGGGCACTGCAGGTG
	p35S::OMTN4-Vc/Vn	OMTN4-Vc/Vn-F	GGGGACTCTAGGATCATGAGCGGGATGAATTCGCTGAGC
		OMTN4-Vc/Vn-F	CTCCATGATCGAATTACTGAGTGAGTTCCACATTTGTGTG
	p35S::OMTN6-Vc/Vn	OMTN6-Vc/Vn-F	GGGGACTCTAGGATCATGAGTTTCATAGGCATGGTGG
		OMTN6-Vc/Vn-F	CTCCATGATCGAATTTTGGGGGTTCCACATCTGTGAC
	p35S::AtCUC1-Vc/Vn	*AtCUC1-Vc/Vn-F*	GGGGACTCTAGGATCATGGATGTTGATGTGTTTAACGGT
		AtCUC1-Vc/Vn-R	CTCCATGATCGAATTGAGAGTAAACGGCCACACAC
	p35S::AtCUC2-Vc/Vn	*AtCUC2-Vc/Vn-F*	GGGGACTCTAGGATCATGGACATTCCGTATTACCAC
		AtCUC2-Vc/Vn-R	CTCCATGATCGAATTGTAGTTCCAAATACAGTCAAGT
	p35S::AtCUC3-Vc/Vn	*AtCUC3-Vc/Vn-F*	GGGGACTCTAGGATCATGATGCTTGCGGTGGAAGAT
		AtCUC3-Vc/Vn-R	CTCCATGATCGAATTCAGCTGGAATCCTAAAGGACAT

Subcellular localization assay	*p35S::OsCUC1-eGFP*	eGFP-OsCUC1-F<br>eGFP-OsCUC1-R	CCCTCGAGATGGAGCGGTGCAGCGTGC<br>ACGCGTCGACGAAGCCCCATGCAAAGGCG
	p35S::OsCUC3-eGFP	*eGFP-OsCUC1-F*<br>eGFP-OsCUC1-R	CCCTCGAGATGCATCATCACTCGGCCACCATGGG<br>ACGCGTCGACTGCGCCCCTGGGCACTGCAGGTG
	p35S::CLD1-eGFP	eGFP-CLD1-F<br>eGFP-CLD1-R	GCTCTAGACATGGGGATGGCCCCCGCCGGTCG<br>TCCCCCGGGCTAGACTTGTAGCCAAATAGCAACAAC
pull-down assay	OsCUC1/*pGEX-6p-1*	GST-OsCUC1 -F<br>GST-OsCUC1 -R	GGGATCCCCGGAATTCATGGAGCGGTGCAGCGTGCT<br>GGCCGCTCGAGTCGACCTAGAAGCCCCATGCAAAGGCG
	CLD1/*pRSFDuet-His*	His-CLD1 -F<br>His-CLD1 -R	CCAGGGATCCGAATTCGAGGAGGAGGAGGACGGCAGCGG<br>ATGCGGCCGCAAGCTTCTATCTGTTCTGGTTGACACCATTT
	OsCUC3/*pRSFDuet-MBP*	MBP-OsCUC3 -F<br>MBP-OsCUC3- R	CCAGGGATCCGAATTCATGCATCATCACTCGGCCACCAT<br>ATGCGGCCGCAAGCTTTCATGCGCCCCTGGGCACTGCAGG
	pCMVTNT^{TM}-GST-OsCUC1	OsCUC1-pTNT-F<br>OsCUC1-pTNT-R	TTGCACTCGAGAATTCATGTCCCCTATACTAGGTTATTGGA<br>GGCCGCCCGGGTCGACCTAGAAGCCCCATGCAAAGGCG
	pCMVTNT^{TM}-MBP-OsCUC3	OsCUC3-pTNT-F<br>OsCUC3-pTNT-R	TTGCACTCGAGAATTCATGAAAATCGAAGAAGGTAAACTGGTA<br>GGCCGCCCGGGTCGACTCATGCGCCCCTGGGCACTGCAGG
	pCMVTNT^{TM}-His-CLD1	CLD1-pTNT-F<br>CLD1-pTNT-R	TTGCACTCGAGAATTCATGGGCAGCAGCCATCACCATCATC<br>GGCCGCCCGGGTCGACCTAGACTTGTAGCCAAATAGCAACAACAAG

Primer sequences for real-time PCR analyses

Gene ID	Gene Name	Sequence (5'→3')
LOC_Os06g23650	*OsCUC1*	Forward primer: TCCTCCACCCGGGATGAGTG<br>Reverse primer: CGAGAAGCACGAGGTGTCGC
LOC_Os08g40030	*OsCUC3*	Forward primer: CGCTCCACCAAGGAGGAATGG<br>Reverse primer: GACCGGAGGAAGGTCATGGAAG
LOC_Os02g36880	*OMTN1*	Forward primer: AGGACACCGGCCTCACGT<br>Reverse primer: GATCCATGCGTTGCTGCC
LOC_Os04g38720	*OMTN2*	Forward primer: TCGCGATCGAGGCATCTAC<br>Reverse primer: CCCAAATCACCAAATAATCTACACCTA
LOC_Os04g38720	*OMTN3*	Forward primer: TGCCTGAATTTGGCAGCTT<br>Reverse primer: ACACCTCACCCCCAATGGA
LOC_Os06g46270	*OMTN4*	Forward primer: ACAATATGGGTAGGGCGATCAA<br>Reverse primer: TCCCTCTTCACATTGCCTTCA
LOC_Os08g10080	*OMTN6*	Forward primer: GGTCGGGAGCTACGAGCAA<br>Reverse primer: TGATGGAGCCCTTCATTGG

qRT-PCR primers for *osa-miR164*

Primer name	Sequence **(5'→3')**
Universal Reverse primer	GTGCAGGGTCCGAGGT
RT primer for *U6*	GTTGGCTCTGGTGCAGGGTCCGAGGTATTCGCACCAGAGCCAACAA ATTT
Forward primer for *U6*	CGCACAAATCGAGAAATGGTCC
RT primer for *osa-miR164c*	GTTGGCTCTGGTGCAGGGTCCGAGGTATTCGCACCAGAGCCAACTG CACG
Forward primer for *osa-miR164c*	CGGCGGTGGAGAAGCAGGGTA

Table S3. Percentages of aberrant florets of *oscuc1*-KO and *oscuc3*-KO plants.

Line	Reduction of stamens	Fusion of filaments
***oscuc1*-KO1**	**96%**	**34%**
**non-KO1^{OsCUC1}**	**0%**	**0%**
***oscuc1*-KO2**	**100%**	**32%**
**non-KO2^{OsCUC1}**	**0%**	**0%**
***oscuc3*-KO1**	**92%**	**100%**
**non-KO1^{OsCUC3}**	**0%**	**0%**
***oscuc3*-KO2**	**6%**	**52%**
**non-KO2^{OsCUC3}**	**0%**	**0%**

For each line, 100 florets were used for the statistic analysis.

Table S4. Percentages of aberrant florets of *omtn4*-KO1 and *omtn6*-KO1 plants.

Line	Reduction of stamens	Fusion of filaments
***omtn4*-KO1**	**4%**	**5%**
**non-KO^{OMTN4}**	**0%**	**0%**
***omtn6*-KO1**	**2%**	**4%**
**non-KO^{OMTN6}**	**0%**	**0%**

For each line, 100 florets were used for the statistic analysis.
